# Sexual Dimorphism in Placental mTORC1 Signaling, Amino Acid Transport, and Mitochondrial Respiration at Term

**DOI:** 10.3390/antiox15080977

**Published:** 2026-08-06

**Authors:** Hiroshi Shimada, Toshihide Sakuragi, Vincent Zaegel, Anita Kramer, Kristen E. Boyle, Theresa L. Powell, Thomas Jansson, Fredrick J. Rosario

**Affiliations:** 1 Department of Obstetrics and Gynecology, University of Colorado Anschutz, Aurora, CO 80045, USA; hshimada.seagreen@gmail.com (H.S.); toshihide.sakuragi@cuanschutz.edu (T.S.); anita.kramer@cuanschutz.edu (A.K.); theresa.powell@cuanschutz.edu (T.L.P.); thomas.jansson@cuanschutz.edu (T.J.); 2 Departments of Obstetrics & Gynecology, Sapporo Medical University, Sapporo 060-8556, Japan; 3 Departments of Obstetrics & Gynecology, University of Occupational and Environmental Health, Kitakyushu 807-8555, Japan; 4 Departments of Pediatrics, Basic Science Research Core, LEAD Center, University of Colorado Anschutz, Aurora, CO 80045, USA; vincentzaegel@gmail.com (V.Z.); kristen.boyle@cuanschutz.edu (K.E.B.); 5 Department of Pediatrics, University of Colorado Anschutz, Aurora, CO 80045, USA

**Keywords:** maternal–fetal exchange, human, fetal growth, nutrient transport, mitochondrial function

## Abstract

Male fetuses generally grow faster in utero and are heavier at birth than females, whereas the rates of perinatal mortality and morbidity are often higher in boys than in girls. Sexual dimorphism in placental function is believed to contribute to these differences. However, evidence for sex-specific differences in placental function remains limited. We hypothesized that placental nutrient-sensing signaling activity, amino acid transport capacity, and mitochondrial respiration are higher in male than female placentas. Placentas were collected from 46 women (BMI 18.5–29.9 kg/m^2^) with uncomplicated pregnancies delivering appropriate-for-gestational-age infants (*n* = 23 female; *n* = 23 male). In homogenates of male placentas, the phosphorylation of S6RP (Ser235/236) was increased and total 4E-BP1 protein expression was reduced compared with female placentas, indicating enhanced mTORC1 signaling. In vitro System A amino acid transport activity was also greater in microvillous plasma membranes isolated from male placentas. Placental mitochondrial respiratory capacity was assessed in villous tissue using high-resolution respirometry with carbohydrate and lipid substrate–uncoupler–inhibitor titration (Carb SUIT and Lipid SUIT) protocols on the Oroboros O2k platform. Male placentas exhibited greater maximal oxidative phosphorylation capacity, enhanced complex II-linked respiration, and higher maximal electron transport system capacity compared with female placentas under both carbohydrate- and lipid-supported respiratory conditions. Although placental weight did not differ between sexes, the birth weight-to-placental weight ratio was significantly higher in male pregnancies, consistent with greater placental functional efficiency. In conclusion, placental mTORC1 signaling activity, System A amino acid transport activity, and mitochondrial respiratory capacity are greater in male than female placentas and are associated with greater placental functional efficiency and fetal growth in male pregnancies.

## 1. Introduction

Sex differences in pregnancy outcomes have been observed for centuries [1]. Specifically, male fetuses generally grow faster than females from the embryonic stage [2,3] to delivery [4,5] and boys are heavier at birth. On the other hand, boys typically have a higher perinatal morbidity and mortality than girls [6,7,8,9]. Male fetuses tend to be at an increased risk for premature rupture of membranes [9,10,11,12], early preterm birth [9,10,12,13], and term preeclampsia [11,14,15], while a female sex of the fetus is associated with a higher incidence of preterm preeclampsia [15,16] and fetal growth restriction (FGR) [10,17] across multiple populations. It is widely believed that sexual dimorphism in placental function contributes to these differences. However, although there is ample evidence that placental gene expression varies with fetal sex [18,19,20,21], information on differences in placental function is more limited.

Mechanistic target of rapamycin (mTOR) is a serine/threonine kinase highly expressed in the syncytiotrophoblast of the human placenta. Trophoblast mTOR signaling has been suggested to function as a placental nutrient sensor, serving as critical hub linking maternal nutrition and metabolism and utero–placental blood flow to placental function, fetal growth, and developmental programming [22]. Trophoblast mTOR signaling responds to an array of diverse maternal nutritional and metabolic signals including circulating levels of hormones, including insulin, IGF-1, and adiponectin, and nutrients, such as amino acids, folate, and fatty acids, to regulate multiple critical placental functions such as amino acid transport [23,24] and mitochondrial respiration [25]. mTOR forms two complexes, mTORC1 and 2 [26,27]. mTORC1 promotes protein translation and cell growth mediated by the phosphorylation of S6 kinase 1 (S6K1) and eukaryotic initiation factor 4E binding protein 1 (4E-BP1) [26]. mTORC2 regulates the actin skeleton, cell cycle progression, anabolism, and cell survival through the phosphorylation of protein kinase B (AKT) and C-α (PKC α) and serum glucocorticoid-regulated kinase 1 (SGK1) [26]. Placental mTORC1 activity has been shown to be linked to fetal growth [28]. Specifically placental mTORC1 activity is decreased in FGR [29,30], while placental mTORC1 activity has been reported to be increased in fetal overgrowth in women with GDM [31] or maternal obesity [32]. Thus, placental mTORC1 activity is positively associated with infant birthweight. mTOR mRNA levels have been reported to be higher in male than female placentas [33]. Moreover, a comparison analysis of two datasets using Ingenuity Pathway Analysis [34,35] demonstrated that the mTOR pathway is upregulated in male first-trimester placentas [36], findings that are in general agreement with a meta-analysis of gene expression data in human placenta revealing that mTOR gene expression is regulated in a sexually dimorphic manner [37]. However, detailed information on fetal sex differences in the activity of placental mTOR signaling in human pregnancy is largely lacking.

Insulin, insulin-like growth factor 1 (IGF-1), and IGF-2 are polypeptides that mainly interact with the type-1 insulin like growth factor receptor (IGF1R) and the insulin receptor (INSR) [38]. When activated, these receptors promote signaling through multiple pathways, including the phospharidylinositol-3 kinase (PI3K)/AKT pathway mediated by insulin receptor substrate 1 (IRS-1), which leads to the activation of mTORC1 and 2 signaling [38]. On the other hand, placental AMPK, which is activated by phosphorylation at Thr-172 in response to an increased AMP/ATP ratio associated with energy deprivation, inhibits mTORC1 [39]. Placental AMPK activity is inversely associated with infant birth weight [32]. Thus, placental IGF-1, AMPK, and mTOR signaling pathways interact to regulate placental function and fetal growth. However, the information on sex differences in placental insulin/IGF-1 and AMPK signaling activity is limited.

O-GlcNAcylation adds O-linked N-acetylglucosamine (O-GlcNAc) to the serine, tyrosine, and threonine residues of intracellular components in an UDP-GlcNAc-dependent manner [40]. UDP-GlcNAc is generated as the end-product substrate from glucose metabolism through the hexosamine biosynthetic pathway, and thus, O-GlcNAcylation is often regarded as a broad marker of the nutrient status [40,41]. Indeed, O-GlcNAc transferase (OGT) is highly expressed in the human placenta [42,43] and involved in multiple signaling pathways, including cellular growth, mitochondrial and endoplasmic reticulum function, and cellular stress [44,45]. Thus, OGT is considered to play an important role in placental function and fetal growth [41,44]. Males have been reported to have lower placental OGT expression than females at term [46]. We recently reported that OGT protein expression in human female placentas is higher in the first and second trimester, but not at term, as compared to male placentas [47]. Moreover, the inhibition of mTORC1 or mTORC2 reduced OGT protein expression in primary human trophoblast cells, demonstrating that mTOR signaling is a positive regulator of placental OGT [47].

Changes in transplacental nutrient transport influence fetal nutrient availability, which determines fetal growth rates and body composition. System A is a sodium-dependent transporter mediating the uptake of non-essential neutral amino acids into the cell [48]. All three known isoforms of System A, SNAT 1 (*SLC38A1*), SNAT 2 (*SLC38A2*), and SNAT 4 (*SLC38A4*), are expressed in the placenta [49]. The placental mRNA expression of *SLC38A1* in mice is lower in females than males [50]. However, whether placental amino acid transport activity differs with fetal sex in women remains to be established.

Most cellular energy is produced through oxidative phosphorylation in mitochondria. Many critical placental functions are energy dependent. In particular, active transport processes in the syncytiotrophoblast, such as the transport of amino acids, calcium, sodium, potassium, and protons all require ATP. Moreover, ATP is required for protein synthesis, and ATP also functions as an important signaling molecule. The placental mitochondrial DNA content and electron transport chain (ETC) complex expression and function have been reported to be altered in several important pregnancy complications such as FGR and preeclampsia [51]. Differences in placental mitochondrial function between females and males may contribute to the difference in fetal growth between sexes. Indeed, in cultured primary human trophoblast cells, TNFα treatment reduces ATP-coupled and maximal mitochondrial respiration in females, but not in males [52]. In addition, male trophoblast cells [53] and male chorionic villi [34] from first trimester placentas are enriched in protein related to mitochondrial function compared to female placentas. However, studies on sexual dimorphism in placental mitochondrial function, such as oxidative phosphorylation, are limited. We hypothesized that placental nutrient-sensing signaling activity, amino acid transport capacity, and mitochondrial respiration are higher in male than female placentas.

## 2. Methods

### 2.1. Study Subjects and Tissue Collection

Informed written consent was obtained from pregnant women recruited to donate their placenta to a data/biorepository for use in multiple research studies. The repository was approved and obtained in accordance with the guidelines of the Institutional Review Board at University of Colorado, Denver (COMIRB 14-1073). Every study subject who was recruited gave written informed consent, and the study protocol was carried out in compliance with the World Medical Association Declaration of Helsinki. De-identified samples and clinical information were provided for use in this study. Placentas were collected from 46 women with uncomplicated pregnancies at term (gestational age, 37–41 weeks) and delivering infants (*n* = 23 female, *n* = 23 male) with normal birth weights (appropriate-for gestational-age; AGA) at University Colorado Hospital. Study subjects had either a normal body mass index or were overweight (BMI, 18.5–29.9 kg/m^2^), before or in early pregnancy. Birth weight z-scores were calculated using the sex- and gestational age-specific Hadlock fetal growth reference [54].

### 2.2. Placental Homogenization

Following delivery, placentas were placed on ice and processed within 10 min. After weighing the placentas, the chorionic plate, fetal membranes, umbilical cord, and decidua were removed, and trophoblast villi were dissected into small pieces of about 1 cm^3^ on ice. These pieces were placed in ice-cold cold Hepes-Tris-buffered saline (0.9% NaCl with 10 mM HEPES-Tris. pH 7.4) and washed a few times with cold 0.9% saline. After the addition of ice-cold Buffer D (250 mM sucrose, 10 mM HEPES, pH 7.4) containing a 1:1000 dilution of protease and phosphatase inhibitors, villous tissues were homogenized using a Polytron Homogenizer (Thomas Scientific, Swedesboro, NJ, USA) at 15,000 rpm for 2 min on ice. Homogenized tissues were placed into tubes, and protease inhibitors and phosphatase inhibitors (1:100) were added. Tubes were frozen in liquid nitrogen and stored at −80 °C until further analyses or processing.

### 2.3. Immunoblotting

Total protein concentrations were determined using the bicinchoninic acid (BCA) protein assay (Thermo Fisher, Waltham, MA, USA). Western blotting was carried out as described previously. Placental homogenates (10 µg protein) were prepared in a 4× Laemmli sample buffer with 2-mercaptoethanol (2-ME) (1 μL of 2-ME per 9 μL of 4× Laemmli) and heated for 5 min at 95 °C, except for blots targeting proteins in the electron transport chain complexes (OXPHOS), for which samples were heated at 37 °C for 10 min before loading. Proteins were separated on 10% precast protein gels (Bio-Rad Laboratories, Hercules, CA, USA) and transferred onto a polyvinylidene fluoride (PVDF) membrane (Bio-Rad Laboratories, Hercules, CA, USA). The membrane was blocked in Tris-buffered saline containing 0.1% Tween 20% (TBS-T) buffer containing either 5% non-fat milk or 5% BSA at room temperature for an hour. Antibodies directed to phosphorylated-S6 ribosomal protein (S6RP) (Ser-235/236), S6RP, phosphorylated-4E-BP1 (Thr-37/46), 4E-BP1, OGT, phosphorylated-AKT (Ser-473 or Thr-308), AKT, phosphorylated AMPK (Thr-172), AMPK, phosphorylated extracellular signal-regulated kinases (ERK)1/2 p44/42 (Thr-202/Tyr-204), and ERK1/2 p44/42 were obtained from Cell Signaling (Boston, MA, USA). An OXPHOS antibody was purchased from Abcam (Cambridge, MA, USA). After blocking, membranes were incubated with primary antibodies at dilution of 1:500–1:1000 in 1 or 5% BSA in TBS-T overnight at 4 °C. After washing with TBS-T, membranes were incubated with an appropriate peroxidase-conjugated secondary antibody at a dilution of 1:3000 with 2 or 5% BSA in TBS-T for an hour at room temperature (Supplemental Table S1). After incubation with secondary antibody and washing with TBS-T, bands were visualized using SuperSignal West Dura Reagent (Thermo Fisher, Waltham, MA, USA). Protein expression of SNAT1, SNAT2, and SNAT4 was determined in isolated microvillous membrane (MVM) vesicles because transporter abundance at the syncytiotrophoblast plasma membrane is a more direct measure of functional transporter availability than expression in whole placental homogenates. Western blotting of SNAT isoforms was carried out as described previously [30]. Briefly, 2 µg of MVM protein was loaded per lane for immunoblot analysis. All band intensities were measured using the G-box system (Syngene, Cambridge, UK). Target band intensities were quantified via densitometry and normalized to protein loading. For all analyses performed using placental homogenates, protein expression was normalized to total protein loading determined through Amido Black staining. In contrast, β-actin was used as the loading control for the analysis of SNAT1, SNAT2, and SNAT4 protein expression in isolated MVM vesicles. Unlike whole placental homogenates, total protein staining is less suitable for MVM preparations because the low protein loading (2 µg per lane) results in weak total protein staining, making it less reliable for normalization. Therefore, β-actin was used as the loading control for MVM preparations. For each protein target, the mean density of the female sample bands was assigned an arbitrary value of 1.0. For selected targets, PVDF membranes were stripped using Thermo Scientific™ Restore™ Western Blot Stripping Buffer (Thermo Fisher Scientific, Waltham, MA, USA, REF46430) and subsequently reprobed with additional primary antibodies. The stripping and reprobing strategy for each membrane is summarized in Supplemental Table S2 and indicated in the corresponding figure legends. Because all samples could not be analyzed on a single membrane, an identical internal reference sample (equalizer) was included on each membrane for every protein target. Band intensities were first normalized to the loading control (Amido Black or β-actin) and subsequently normalized to the internal reference sample to account for inter-membrane variability before combining data from multiple membranes for statistical analysis.

### 2.4. Activity of Amino Acid Transporters

For the System A amino acid transport activity and SNAT isoform expression analyses, a subset of placentas (*n* = 9–10) was randomly selected from the larger signaling cohort (Supplemental Table S3). Syncytiotrophoblast MVMs were isolated according to a well-characterized protocol [55] that we have used extensively [30]. The isolated MVM vesicles were stored at −80 °C in buffer containing protease and phosphatase inhibitors until studies of nutrient transporter activity. MVM enrichment was assessed using the MVM/homogenate ratio of alkaline phosphatase activity. MVM enrichment was similar in MVMs isolated from female (16.9 ± 2.03) and male placentas (14.0 ± 1.32, *p* = 0.24). The uptake of MVM amino acids (mediated by System A) was measured using standard rapid filtration techniques as described previously [30]. Briefly, vesicles were preloaded in vesicle loading buffer (10 mM Hepes-Tris, 300 mM mannitol, pH 7.4) overnight at 4 °C. On the next day, the vesicles were centrifuged (30 min, 4 °C, at 125,000× *g*), resuspended in the appropriate volume of vesicle loading buffer, and placed on ice. Vesicles were allowed to equilibrate to 37 °C for 10 to 15 min before starting transport experiments. At time zero, 30 μL of vesicles was rapidly mixed with incubation buffer (150 mM NaCl, 10 mM Hepes–Tris, pH 7.4, 37 °C) containing ^14^C-methyl-aminoisobutyric acid (MeAIB, 150 μM). After 20 s (s), the uptake of MeAIB was stopped by adding 2 mL of ice-cold PBS and rapidly separating vesicles from the substrate medium via filtration on mixed ester filters (0.45 μm pore size, Millipore Corporation, Burlington, MA, USA), and samples were washed three times with 2 mL of PBS. Filters were dissolved in 2 mL of liquid scintillation fluid and counted. The uptake was expressed as pmol/(mg protein × 20 s). The Na^+^-dependent uptake of MeAIB was calculated by subtracting Na^+^-independent uptakes (using Na^+^ free buffers containing KCl instead of NaCl) from total uptakes.

### 2.5. Measurement of Mitochondrial Oxidative Respiratory Capacity

For the mitochondrial respiratory capacity analyses, placental villous tissue was obtained from a separate cohort specifically recruited for high-resolution respirometry experiments (Supplemental Table S4). Mitochondrial oxidative respiratory capacity was assessed in small pieces of villous tissue (approximately 30–40 mg), which were placed in 2 mL Nunc Tubes with 250 μL of cryopreservation solution (0.21 M mannitol, 0.07 M sucrose, made up in 30% DMSO, 70% ddH_2_O, pH 7.5) for 30 s and subsequently rapidly frozen in liquid nitrogen in accordance with established protocols [56,57,58]. Mitochondrial respiratory capacity was measured using a high-resolution respirometer (Oxygraph-2k, Oroboros Instruments, Innsbruck, Austria). Frozen tissues samples were placed in thawing medium (0.25 M sucrose, 0.01 M TRIS-HCL, pH 7.5) at a ratio of 4:1 (medium:tissue) for 20 s in a 45 °C water bath. Thawed tissue samples were gently dissected using a microscope to remove large fibrous vessel branches and retain terminal villi. During this dissection, the tissue was kept in chilled biopsy preservation medium (BIOPS) (2.77 mM CaK_2_EGTA, 7.2 mM K_2_EGTA, 5.77 mM Na_2_ATP, 6.56 mM MgCl_2_.6H_2_O, 20 mM taurine, 15 mM Na_2_phosphocreatine, 20 mM imidazole, 0.5 mM dithiothreitol, 50 mM MES hydrate, pH 7.1). Subsequently the tissue was permeabilized with 50 μg/mL of saponin in 1 mL BIOPS for 20 min with gently shaking at 4 °C and transferred to respiratory medium (Mir05) (0.5 mM EGTA, 3 mM MgCl2.6H2O, 60 mM K-lactobionate, 20 mM taurine, 10 mM KH2PO4, 20 mM HEPES, 110 mM sucrose, 1 mg/mL fatty acid free bovine serum albumin, pH 7.1) for washing 3 times at 4 °C [57]. Each chamber of the Oxygraph-2k respirometer was closed to increase O_2_ from the atmospheric concentration (150 nmol/mL) to between 300 and 350 nmol/mL, and each chamber was kept at 37 °C. DatLab software (V7, Oroboros Instruments, Innsbruck, Austria) was used for real-time oxygen concentration and consumption acquisition and data analysis. Respiratory rates were expressed as oxygen consumption per mg of villous tissue.

The activity of specific respiratory states was determined by the addition of substrates and electron transport system (ETS) inhibitors as previously described [56,58]. The Carb (carbohydrate) Substrate Uncoupler Inhibitor Titrations (SUIT) protocol was used to assess the tricarboxylic acid cycle. The Carb SUIT protocol was measured following the addition of malate (2 mM) and pyruvate (5 mM) to determine complex I-linked respiration in the absence of ATP synthesis (PM*_L_*; LEAK respiration) in the nicotinamide adenine dinucleotide (NADH) pathway. ADP (10 mM) was added to activate the phosphorylation of ADP to ATP through complex I (PM*_P_*; complex I OXPHOS). Glutamate (10 mM) was added (GMP*_P_*) followed by cytochrome C (10 μM), which was used as quality control to confirm outer mitochondrial membrane integrity after the addition of ADP and glutamate, with data excluded if respiration increased by >30% [56,58]. Succinate (10 mM) was added to achieve a maximum increase in the oxygen consumption rate (GMS*_P_*; maximal OXPHOS). Complex I was inhibited through the addition of rotenone (1 μM), restricting electron flux to the S-pathway via complex II (S*_E_*). The uncoupler carbonyl cyanide p-trifluoro methoxyphenyl hydrazone (FCCP) (1 μM) was then added to measure maximum respiratory capacity (GMS*_E_*). FCCP was titrated incrementally to determine maximal electron transport system (ETS) capacity (GMS_E_). Finally, antimycin A (5 μM) was added to inhibit complex III to determine residual oxygen consumption (ROX), which was measured.

The Lipid SUIT protocol was used to assess mitochondrial β-oxidation. The Lipid SUIT was measured following the addition of palmitoyl carnitine (5 μM) and malate (1 mM) to determine complex I-linked respiration (PalM*_L_*; β-oxidation LEAK respiration) in the fatty acid oxidative (FAO) pathway. ADP (10 mM) was added to activate the phosphorylation of ADP to ATP (PalM*_P_*; β-oxidation OXPHOS) followed by the addition of cytochrome C (10 μM), which was used as quality control to confirm the outer mitochondrial membrane integrity [58]. Glutamate (10 mM) (GMP*_P_*) was sequentially added followed by succinate (10 mM) to achieve a maximum increase in the oxygen consumption rate (GMS*_P_*; maximum OXPHOS). Subsequent additions were identical to the Carb SUIT.

### 2.6. Statistical Analysis

The number of replicates (*n*) represents the number of individual placentas studied. Datasets were assessed for normality using the Shapiro–Wilk normality test. For normally distributed data, statistical differences between female and male placentas were evaluated using an unpaired Student’s *t*-test. For non-normally distributed data, the Mann–Whitney test was used. Datasets that were not normally distributed are identified in the corresponding figure legends. Potential outliers were identified using Grubbs’ test (α = 0.05). For placental signaling proteins and mitochondrial electron transport chain protein expression analyses, identified outliers were excluded from the final statistical analyses because of the relatively large sample size (*n* = 46). In contrast, for System A amino acid transport activity, SNAT isoform expression, and mitochondrial respiration (Oroboros) analyses, identified outliers were retained in the statistical analyses because of the smaller sample sizes, thereby preserving statistical power. Details of all identified outliers and whether they were retained or excluded are provided in the corresponding figure legends and supplemental tables. Data are presented as means ± SEMs. Statistical analyses were performed using Prism 11.0.1 (GraphPad Software, Boston, MA, USA). A *p* value < 0.05 was considered statistically significant.

## 3. Results

### 3.1. Clinical Characteristics

The clinical characteristics of the study participants are summarized in Table 1. The maternal age (*p* = 0.11), pre-pregnancy BMI (*p* = 0.34), number of women with overweight (*p* = 0.67), gestational age at delivery (*p* = 0.47), placental weight (*p* = 0.54), and mode of delivery (*p* = 1.00) did not differ significantly between pregnancies with female and male fetuses. Male infants had a significantly greater birth weight compared with female infants (3401 ± 65.7 g vs. 3209 ± 57.7 g, *p* = 0.03). Birth weight z-scores, calculated using the sex- and gestational age-specific Hadlock fetal growth reference [54], were comparable between groups (*p* = 0.78), indicating that both male and female infants exhibited fetal growth appropriate for their gestational age and sex. Although the placental weight did not differ between female and male pregnancies, the birth weight-to-placental weight ratio was significantly higher in male pregnancies than in female pregnancies (5.17 ± 0.13 vs. 5.77 ± 0.23, *p* = 0.03), indicating greater fetal weight supported per gram of placental tissue.

### 3.2. Higher S6RP Phosphorylation and Lower Total 4E-BP1 Protein Expression in Male Placentas

**Figure 1:**
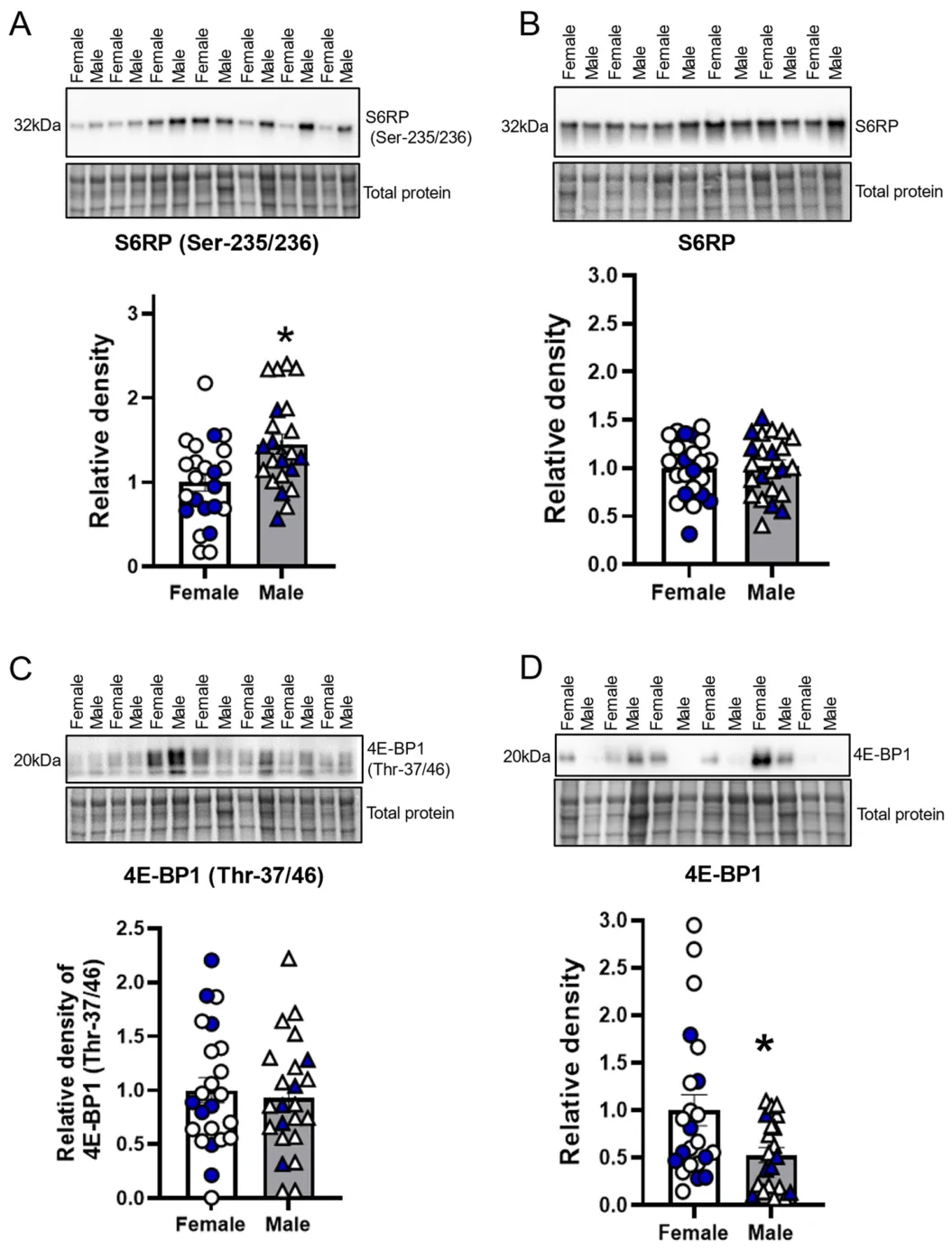
Placental mTORC1 signaling in female and male placentas. Representative Western blots and quantification of Western blot analyses for phosphorylated S6RP (Ser-235/236) (**A**), total S6RP (**B**), phosphorylated 4E-BP1 (Thr-37/46) (**C**), total 4E-BP1 (**D**) in homogenates from female and male term placentas. Histogram bars summarize densitometric analyses of Western blots. For each protein target, the mean density of female sample bands was assigned an arbitrary value of 1.0. Data are presented as means ± SEMs. * *p* < 0.05 versus female placentas. Statistical analyses were performed using an unpaired Student’s *t*-test or Mann–Whitney test. One outlier was identified in the phosphorylated S6RP (Ser235/236) analysis, and two outliers were identified in the total 4E-BP1 analysis using Grubbs’ test (α = 0.05); all identified outliers were male samples. These outliers were removed from statistical analyses. Final sample sizes were *n* = 21–23 female/male placentas. All datasets were assessed for normality using the Shapiro–Wilk normality test. Data shown in panels (**A**–**C**) were normally distributed, and statistical differences were evaluated using an unpaired Student’s *t*-test. Data shown in panel (**D**) were not normally distributed and were analyzed using the Mann–Whitney test. Abbreviations: S6RP, S6 ribosomal protein; 4E-BP1, eukaryotic initiation factor 4E-binding protein 1; Ser, Serine; Thr, Threonine. Following the detection of phospho-4E-BP1 (Thr-37/46), the membrane was stripped and reprobed with an antibody against phospho-S6RP (Ser-235/236). Details of the stripping and reprobing strategy are provided in Supplemental Table S2. To illustrate the distribution of delivery modes within each sex, samples from vaginal deliveries are highlighted in blue, whereas white symbols indicate placentas delivered via cesarean section. The expected molecular weights of phosphorylated S6RP (Ser-235/236) and total S6RP are 32 kDa, whereas phosphorylated 4E-BP1 (Thr-37/46) and total 4E-BP1 are approximately 20 kDa. Full-length, uncropped Western blot images with molecular weight markers are provided in the Supplemental Material.

S6RP and 4E-BP1 were measured as key downstream effectors of mTORC1 signaling that regulate protein synthesis, cellular growth, and placental nutrient-sensing activity. The phosphorylation of S6RP reflects the activation of translational machinery and cell growth pathways, whereas the phosphorylation-mediated inhibition of 4E-BP1 promotes cap-dependent mRNA translation. Together, these targets provide functional indicators of mTORC1 activity and placental anabolic signaling. Male placentas exhibited significantly greater activation of mTORC1 downstream signaling compared with female placentas. Specifically, the phosphorylation of S6 ribosomal protein (S6RP) at Ser-235/236 was 45% higher in male placentas than in female placentas (*p* = 0.006; Figure 1A). Total S6RP protein expression was comparable between female and male placentas (*p* = 0.78; Figure 1B). Phosphorylated 4E-BP1 (Thr37/46) protein expression was comparable between female and male placentas (*p* = 0.88; Figure 1C). In contrast, total 4E-BP1 protein expression was 48% lower in male placentas relative to female placentas (*p* = 0.03; Figure 1D), consistent with the reduced inhibitory restraint on mTORC1 signaling and therefore greater pathway activation in male placentas. Consistent with these findings, the ratio of phosphorylated 4E-BP1 (Thr-37/46) to total 4E-BP1 expression was significantly elevated in male placentas (+ 109%, *p* = 0.03), indicating greater relative 4E-BP1 phosphorylation (Supplemental Figure S1A). Similarly, the ratio of phosphorylated S6RP (Ser-235/236) to total S6RP expression was 48% higher in male placentas compared with female placentas (*p* = 0.03) (Supplemental Figure S1B). All outliers identified in the placental signaling pathway analyses are summarized in Supplemental Table S5.

**Figure 2:**
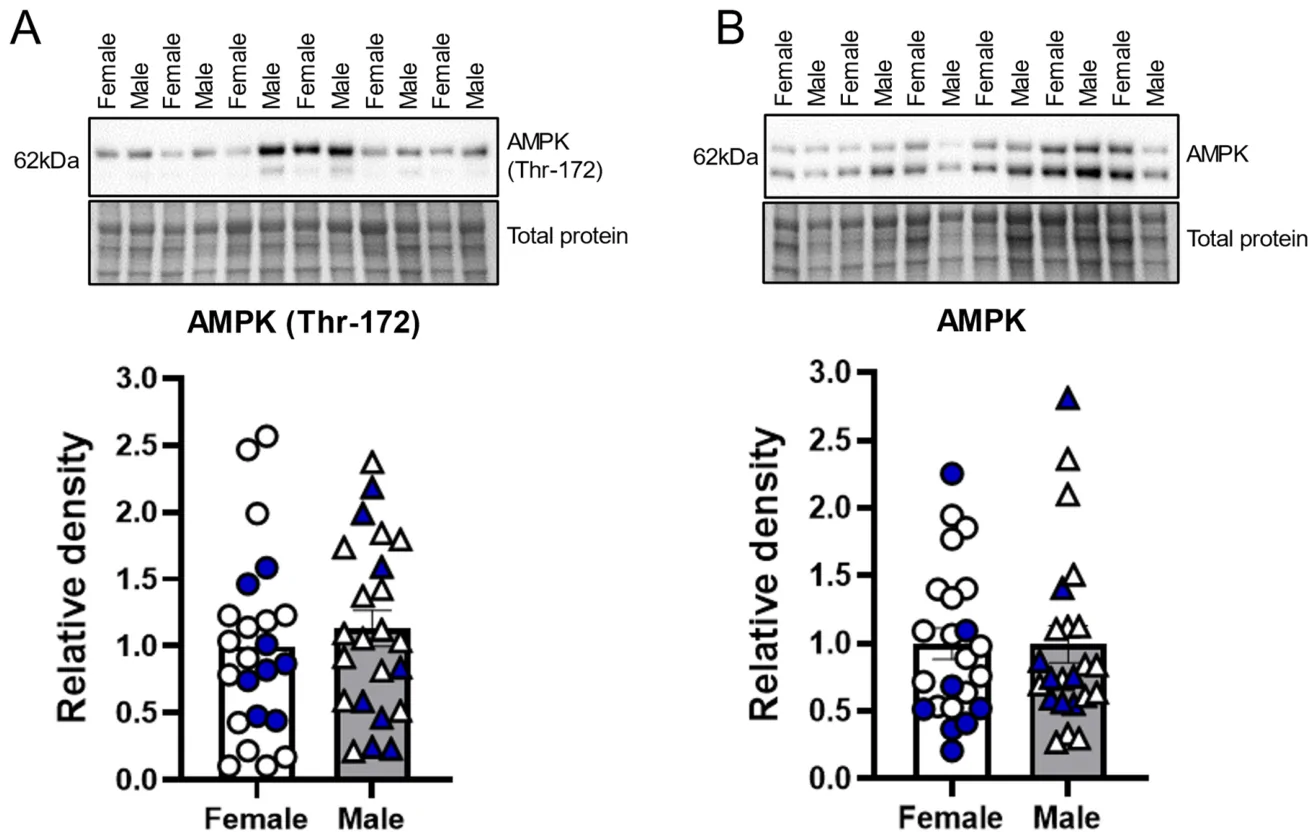
Placental AMPK signaling in female and male placentas. Representative Western blots and quantification of Western blot analyses for phosphorylated AMPK (Thr-172) (**A**) and total AMPK (**B**) in homogenates from female and male term placentas. Histogram bars summarize densitometric analyses of Western blots. For each protein target, the mean density of female sample bands was assigned an arbitrary value of 1.0. Data are presented as means ± SEMst. Statistical analyses were performed using an unpaired Student’s *t*-test or Mann–Whitney test. One outlier was removed from total AMPK analyses using Grubbs’ test (α = 0.05); all identified outliers were male samples. Final sample sizes were *n* = 21–22 female placentas and *n* = 22–23 male placentas depending on the analysis. Data shown in panel (**A**) were normally distributed, and statistical differences were evaluated using an unpaired Student’s *t*-test. Data shown in panel (**B**) were not normally distributed and were analyzed using the Mann–Whitney test. Abbreviations: AMPK, 5′ AMP-activated protein kinase; Thr, Threonine. To illustrate the distribution of delivery modes within each sex, samples from vaginal deliveries are highlighted in blue, whereas white symbols indicate placentas delivered via cesarean section. The molecular weight marker (kDa) is indicated on the left of each representative immunoblot. The expected molecular weights of phosphorylated AMPK (Thr172) and total AMPK are 62 kDa. Full-length, uncropped Western blot images including molecular weight markers are provided in the Supplemental Material.

AMPK was investigated as a key cellular energy sensor that regulates metabolic homeostasis, nutrient availability, and placental adaptation to energetic stress. Because AMPK signaling interacts with mTOR pathways and can influence placental growth and nutrient transport, assessing AMPK activity helped determine whether sex-specific differences in placental signaling extended to energy-sensing pathways. However, no significant sex differences were observed in AMPK signaling in the placenta. Protein expression of phosphorylated AMPK (Thr-172, *p* = 0.50) and total AMPK (*p* = 0.80) did not differ between female and male placentas (Figure 2A,B). The ratio of phosphorylated AMPK (Thr-172) to total AMPK protein expression was comparable (*p* = 0.08) between male and female placentas (Supplemental Figure S2A).

**Figure 3:**
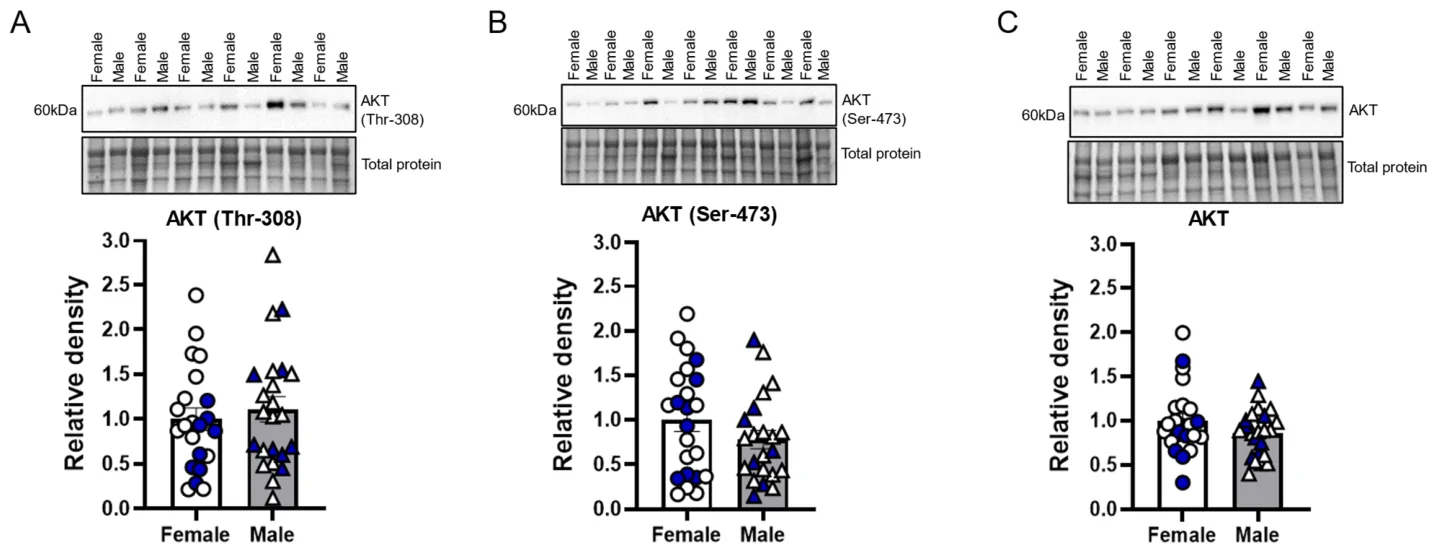
Placental AKT signaling in female and male placentas. Representative Western blots and quantification of Western blot analyses for phosphorylated AKT at Thr-308 (**A**), phosphorylated AKT at Ser-473 (**B**), and total AKT protein expression (**C**) in homogenates from female and male term placentas. Histogram bars summarize densitometric analyses of Western blots. For each protein target, the mean density of female sample bands was assigned an arbitrary value of 1.0. Data are presented as means ± SEMs. Statistical analyses were performed using an unpaired Student’s *t*-test. One outlier was removed from phosphorylated AKT-Thr-308 analyses (female sample) and one outlier was removed from phosphorylated AKT Ser-473 analyses (male sample) using Grubbs’ test (α = 0.05). No outliers were identified for total AKT expression. Final sample sizes were *n* = 22–23 female placentas and *n* = 22–23 male placentas depending on the analysis. All data were normally distributed as assessed via the Shapiro–Wilk normality test. Abbreviations: AKT, protein kinase B; Ser, Serine; Thr, Threonine. To illustrate the distribution of delivery modes within each sex, samples from vaginal deliveries are highlighted in blue, whereas white symbols indicate placentas delivered by cesarean section. The molecular weight marker (kDa) is indicated on the left of each representative immunoblot. The expected molecular weights of phosphorylated AKT (Thr-308), phosphorylated AKT (Ser-473), and total AKT are 60 kDa. Full-length, uncropped Western blot images including molecular weight markers are provided in the Supplemental Material.

AKT was investigated because it is a major upstream regulator of mTOR signaling and plays a central role in placental growth, metabolism, nutrient sensing, and cell survival. The phosphorylation of AKT at Thr-308 and Ser-473 reflects pathway activation and can regulate downstream anabolic signaling, including S6RP and 4E-BP1 activity. An assessment of AKT signaling determined whether sex-specific differences in placental mTORC1 activation were associated with alterations in upstream insulin/growth factor signaling pathways. However, no significant sex differences were observed in AKT signaling in the placenta. Specifically, the protein expression of phosphorylated AKT at Thr-308 (*p* = 0.56) and AKT at Ser-473 (*p* = 0.19), as well as total AKT (*p* = 0.17) protein expression, did not differ between female and male placentas (Figure 3A–C). The ratios of phosphorylated AKT (Thr-308, *p* = 0.25) to total AKT and phosphorylated AKT (Ser-473, *p* = 0.93) to total AKT protein expression were comparable between male and female placentas (Supplemental Figure S2B,C).

### 3.3. No Difference in Protein Expression of OGT or ERK 1/2 Between Female and Male Placentas

**Figure 4:**
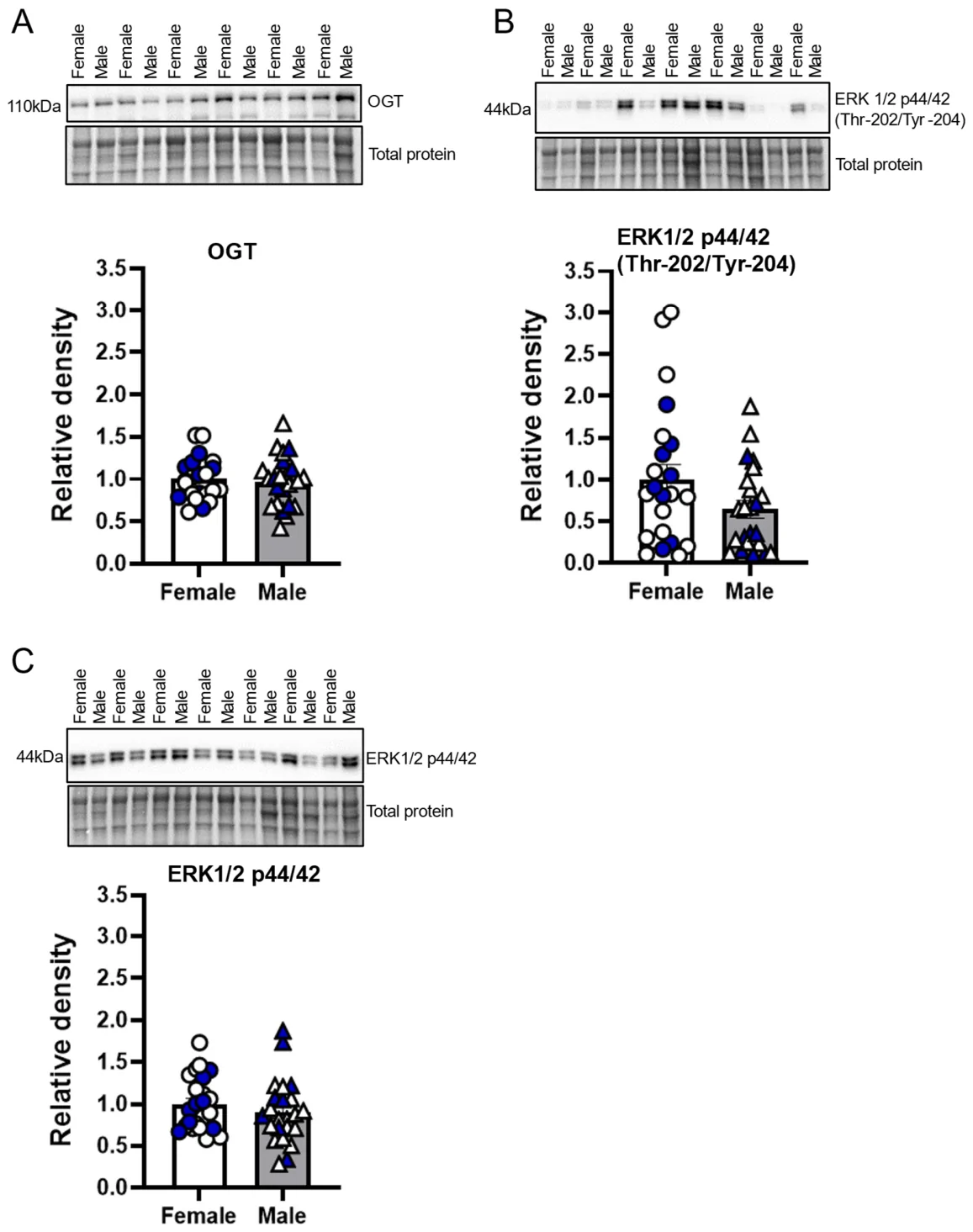
Placental OGT and ERK1/2 signaling in female and male placentas. Representative Western blots and quantification of Western blot analyses for OGT protein expression (**A**), phosphorylated ERK1/2 p44/42 at Thr-202/Tyr-204 (**B**), and total ERK1/2 p44/42 protein expression (**C**) in homogenates from female and male term placentas. Histograms summarize densitometric analyses of Western blots. For each protein target, the mean density of female sample bands was assigned an arbitrary value of 1.0. Data are presented as means ± SEMs. Statistical analyses were performed using an unpaired Student’s *t*-test or Mann–Whitney test. No outliers were identified using Grubbs’ test (α = 0.05). Final sample sizes were *n* = 23 female placentas and *n* = 23 male placentas. Data shown in panels (**A**,**C**) were normally distributed, and statistical differences were evaluated using an unpaired Student’s *t*-test. Data shown in panel (**B**) were not normally distributed and were analyzed using the Mann–Whitney test. Abbreviations: OGT, O-GlcNAc transferase; ERK, extracellular signal-regulated kinase; Thr, Threonine; Tyr, tyrosine. Following the detection of phospho-AKT (Thr-308), the PVDF membrane was stripped using Thermo Scientific™ Restore™ Western Blot Stripping Buffer and subsequently reprobed with an antibody against OGT. Details of the stripping and reprobing strategy are provided in Supplemental Table S2. To illustrate the distribution of delivery modes within each sex, samples from vaginal deliveries are highlighted in blue, whereas white symbols indicate placentas delivered via cesarean section. The molecular weight marker (kDa) is indicated on the left of each representative immunoblot. The expected molecular weights of OGT (110 kDa), phosphorylated ERK1/2 p44/42 (Thr202/Tyr204) (44 kDa), and total ERK1/2 p44/42 (44 kDa) are shown. Full-length, uncropped Western blot images including molecular weight markers are provided in the Supplemental Material.

OGT and ERK1/2 signaling pathways were examined because both are involved in placental growth, nutrient sensing, cellular stress responses, and trophoblast function. OGT is a nutrient-sensitive enzyme that regulates protein O-GlcNAcylation and has been implicated in sex-specific placental adaptations to environmental and metabolic stress. ERK1/2 is a key component of the MAPK signaling pathway and regulates cell proliferation, differentiation, and survival, while also interacting with mTOR-related signaling pathways. Assessing these targets helped determine whether sex-specific differences in placental signaling extended beyond mTORC1 signaling pathways. However, no significant sex differences were observed in placental OGT or ERK1/2 signaling. Protein expression of OGT did not differ between female and male placentas (*p* = 0.71; Figure 4A). Similarly, there were no differences in phosphorylated ERK1/2 p44/42 (Thr-202/Tyr-204, *p* = 0.19) or total ERK1/2 p44/42, *p* = 0.37) protein expression between female and male placentas (Figure 4B,C). The ratio of phosphorylated ERK1/2 p44/42 (Thr-202/Tyr-204) to total ERK1/2 p44/42 protein expression was comparable (*p* = 0.93) between male and female placentas (Supplemental Figure S2D).

### 3.4. Higher Activity of System a Amino Acid Transporters in Male MVM Vesicles

**Figure 5:**
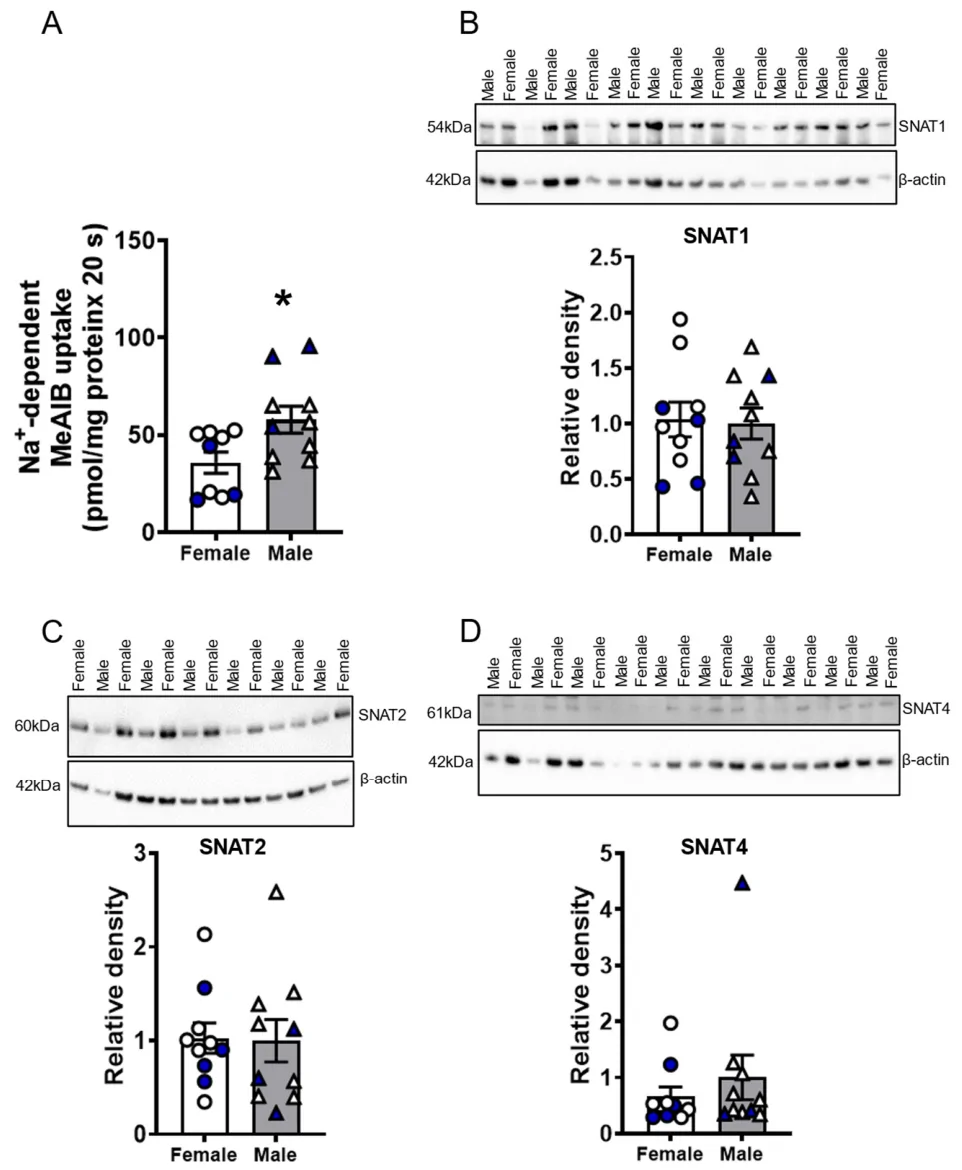
System A amino acid transport activity and SNAT transporter protein expression in placental MVM vesicles from female and male placentas. Na+-dependent MeAIB uptake measured at 20 s in microvillous membrane (MVM) vesicles isolated from female (*n* = 9) and male (*n* = 10) term placentas (**A**). The initial rate of uptake is expressed as pmol/mg × 20 s. Representative Western blots and quantification of Western blot analyses for SNAT1 (**B**), SNAT2 (**C**), and SNAT4 (**D**) protein expression in MVM vesicles isolated from female and male placentas. Histograms summarize densitometric analyses of Western blots. For each protein target, the mean density of female sample bands was assigned an arbitrary value of 1.0. Data are presented as means ± SEMs. * *p* < 0.05 versus female placentas. Statistical analyses were performed using an unpaired Student’s *t*-test or Mann–Whitney test. No outliers were identified for Na+-dependent MeAIB uptake, SNAT1, or SNAT2 analyses. Two outliers (one female and one male sample) were identified in the SNAT4 analyses using Grubbs’ test (α = 0.05) and retained in the final statistical analysis. Final sample sizes were *n* = 9–10 per group on the analysis. Data shown in panels (**B**,**C**) were normally distributed, and statistical differences were evaluated using an unpaired Student’s *t*-test. Data shown in panels (**A**,**D**) were not normally distributed and were analyzed using the Mann–Whitney test. All data were normally distributed as assessed via the Shapiro–Wilk normality test. Abbreviations: MVM, microvillous membrane; MeAIB, methylaminoisobutyric acid; SNAT, sodium-coupled neutral amino acid transporter; sec, second; *n*, number. To illustrate the distribution of delivery modes within each sex, samples from vaginal deliveries are highlighted in blue, whereas white symbols indicate placentas delivered via cesarean section. The molecular weight marker (kDa) is indicated on the left of each representative immunoblot. The expected molecular weights of SNAT1, SNAT2, SNAT4, and β-actin are 54, 60, 61, and 42 kDa, respectively. Full-length, uncropped Western blot images including molecular weight markers are provided in the Supplemental Material.

System A amino acid transport was investigated because placental amino acid transport is a key determinant of the fetal nutrient supply and growth. To assess placental amino acid transport activity, a subset of samples from the signaling cohort was randomly selected (*n* = 10 female; *n* = 10 male; Supplemental Table S3). A time-course experiment confirmed that the 20 s time point represented initial-rate uptake conditions for transport measurements (Supplemental Figure S3). Microvillous membrane (MVM) System A amino acid transport activity was significantly higher in male placentas compared with female placentas, with sodium-dependent MeAIB uptake 52% higher in male MVM vesicles (*p* = 0.04; Figure 5A). Despite the increase in System A transport activity, there were no significant sex differences in the protein expression of SNAT1 (*p* = 0.87; Figure 5B), SNAT2 (*p* = 0.93; Figure 5C), or SNAT4 (*p* = 0.53; Figure 5D) in MVMs isolated from male and female placentas. These findings suggest that the greater amino acid transport activity observed in male placentas is unlikely to be explained by altered transporter abundance.

### 3.5. Increased Maximal Mitochondrial Oxidative Capacity in Male Placentas During Carbohydrate-Supported Respiration

Villous tissue was collected from 20 term placentas (*n* = 12 female, *n* = 8 male; Supplemental Table S4) for the assessment of mitochondrial oxidative respiratory capacity using high-resolution respirometry. The placental mitochondrial respiratory function was evaluated using the carbohydrate substrate–uncoupler–inhibitor titration (Carb SUIT) protocol to examine substrate-specific respiration and maximal electron transport system (ETS) capacity in placental villous tissue.

**Figure 6:**
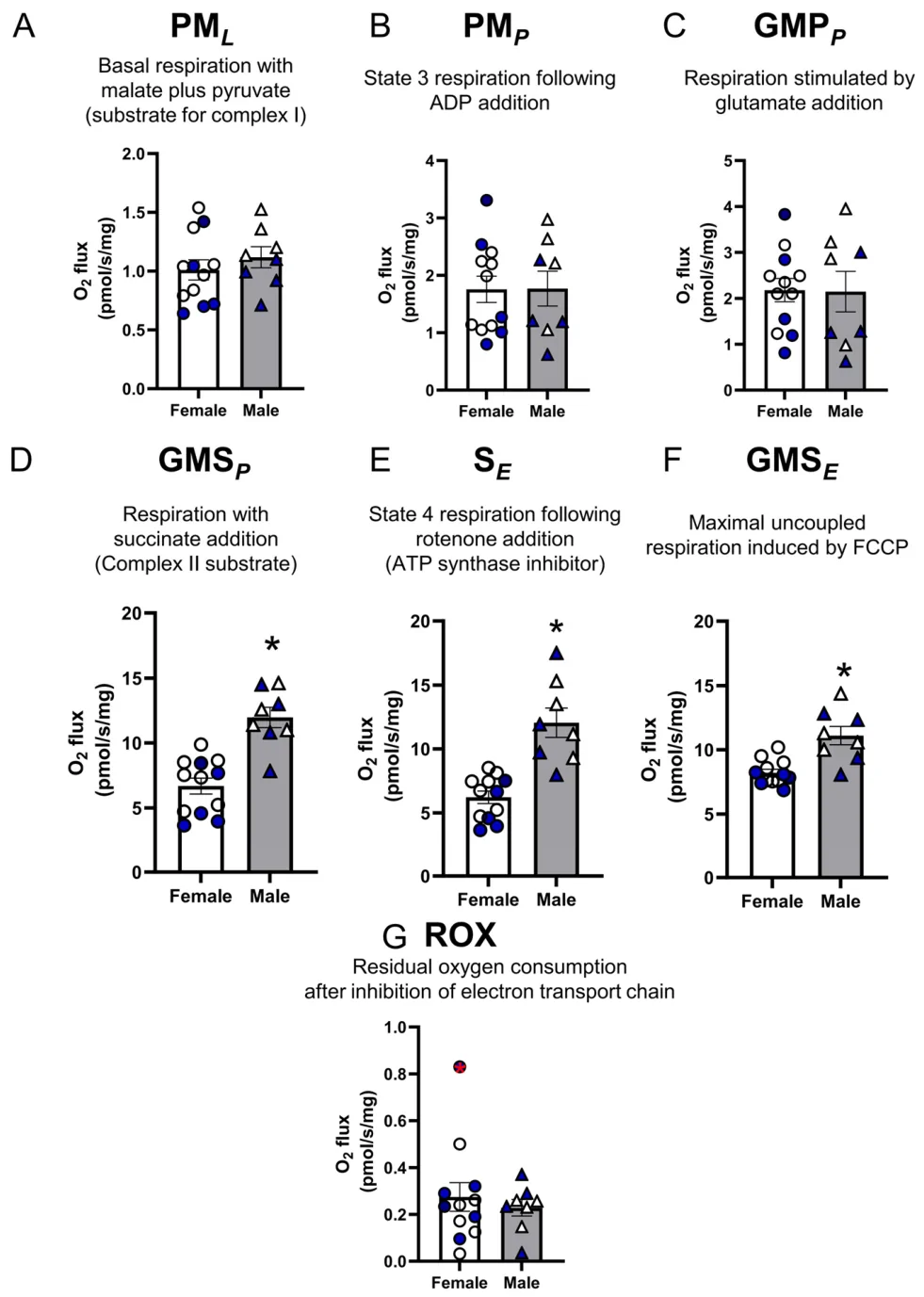
Placental mitochondrial respiratory capacity assessed using the Carbohydrate SUIT protocol in female and male placentas. Mitochondrial respiratory function in term placental villous tissue was measured using the carbohydrate substrate–uncoupler–inhibitor titration (Carb SUIT) protocol and expressed as oxygen flux normalized to placental wet weight. LEAK respiration supported by malate and pyruvate (PM*_L_*) (**A**); ADP-stimulated oxidative phosphorylation through complex I with malate and pyruvate (PM*_P_*) (**B**); glutamate-supported complex I oxidative phosphorylation (GMP*_P_*) (**C**); maximal oxidative phosphorylation capacity following succinate addition, reflecting convergent complex I and II electron flow (GMS*_P_*) (**D**); complex II-supported respiration following rotenone inhibition of complex I (S*_E_*) (**E**); maximal uncoupled respiratory capacity induced by FCCP (GMS*_E_*) (**F**); and residual oxygen consumption following inhibition of the electron transport chain with antimycin A (ROX) (**G**). Data are presented as means ± SEMs. * *p* < 0.05 versus female placentas. No outliers were identified for PM*_L_*, PM*_P_*, GMP*_P_*, GMS*_P_*, S*_E_*, or GMS*_E_* analyses. One outlier (female sample) was identified in the ROX analyses using Grubbs’ test (α = 0.05); however, the sample was retained in the final analysis. Final sample sizes were *n* = 12 female placentas and *n* = 8 male placentas depending on the analysis. All data were normally distributed as assessed via the Shapiro–Wilk normality test, with the exception of PalM_L_, which was analyzed using the Mann–Whitney test. Statistical differences for all other analyses were evaluated using an unpaired Student’s *t*-test. Abbreviations: PM*_L_*, pyruvate/malate LEAK respiration; PM*_P_*, pyruvate/malate oxidative phosphorylation; GMP*_P_*, glutamate-supported oxidative phosphorylation; GMS*_P_*, glutamate/malate/succinate oxidative phosphorylation; S*_E_*, succinate-supported respiration; GMS*_E_*, maximal uncoupled electron transport system capacity; FCCP, carbonyl cyanide-p-trifluoromethoxy phenylhydrazone; ROX, residual oxygen consumption. To illustrate the distribution of delivery modes within each sex, samples from vaginal deliveries are highlighted in blue, whereas white symbols indicate placentas delivered by cesarean section. The red asterisk indicates an outlier, included in the statistical analysis.

In the Carb SUIT protocol, no significant sex differences were observed in NADH-linked LEAK respiration supported by pyruvate and malate (PM*_L_*; *p* = 0.41; Figure 6A). Similarly, there were no significant differences between female and male placentas in complex I-supported oxidative phosphorylation following ADP addition (PM*_P_*) or glutamate stimulation (GMP*_P_*) (*p* = 0.97, *p* = 0.94, respectively; Figure 6B,C). In contrast, the maximal oxidative phosphorylation capacity following succinate addition (GMS*_P_*), reflecting convergent electron input through complexes I and II, was 79% higher in male placentas compared with female placentas (*p* = 0.0001; Figure 6D). Moreover, following rotenone inhibition of complex I, succinate-driven complex II respiration (S*_E_*) was also increased by 94% in male placentas (*p* = 0.0001; Figure 6E), suggesting enhanced complex II-linked respiratory activity. Furthermore, FCCP-induced maximal uncoupled respiration (GMS*_E_*), representing maximal ETS capacity, was 35% greater in male placentas relative to female placentas (*p* = 0.0004; Figure 6F). Together, these findings indicate enhanced mitochondrial oxidative capacity in male placentas despite no differences in basal or complex I-linked respiration. Residual oxygen consumption measured following inhibition of the ETS (ROX) was not significantly different between female and male placentas (*p* = 0.93; Figure 6G), indicating similar levels of non-mitochondrial oxygen consumption. Collectively, these findings demonstrate greater maximal mitochondrial oxidative capacity in male placentas, particularly in pathways involving complex II-supported respiration and maximal ETS function.

To determine whether the observed sex differences in mitochondrial respiration reflected intrinsic differences in mitochondrial function, flux control ratios (FCRs) were calculated by normalizing each respiratory state to the maximal electron transport system (ETS) capacity. No significant sex differences were observed in the FCRs for basal LEAK respiration supported by pyruvate and malate (PM_L_; *p* = 0.11), ADP-stimulated complex I oxidative phosphorylation (PM_P_; *p*= 0.14), or glutamate-supported complex I respiration (GMP_P_; *p* = 0.15) (Supplemental Figure S4A–C). In contrast, male placentas exhibited significantly higher (+30%) FCRs for maximal oxidative phosphorylation following succinate addition (GMS_P_; *p* = 0.03) and for succinate-supported complex II respiration (+30%) following rotenone inhibition (S_E_; *p* = 0.04) compared with female placentas (Supplemental Figure S4D–E). These findings indicate that, in addition to higher absolute respiratory capacity, male placental mitochondria exhibit a greater relative contribution of convergent complex I/II oxidative phosphorylation and complex II-supported respiration to maximal ETS capacity.

### 3.6. Enhanced Maximal Mitochondrial Oxidative Capacity in Male Placentas During Fatty Acid-Supported Respiration

**Figure 7:**
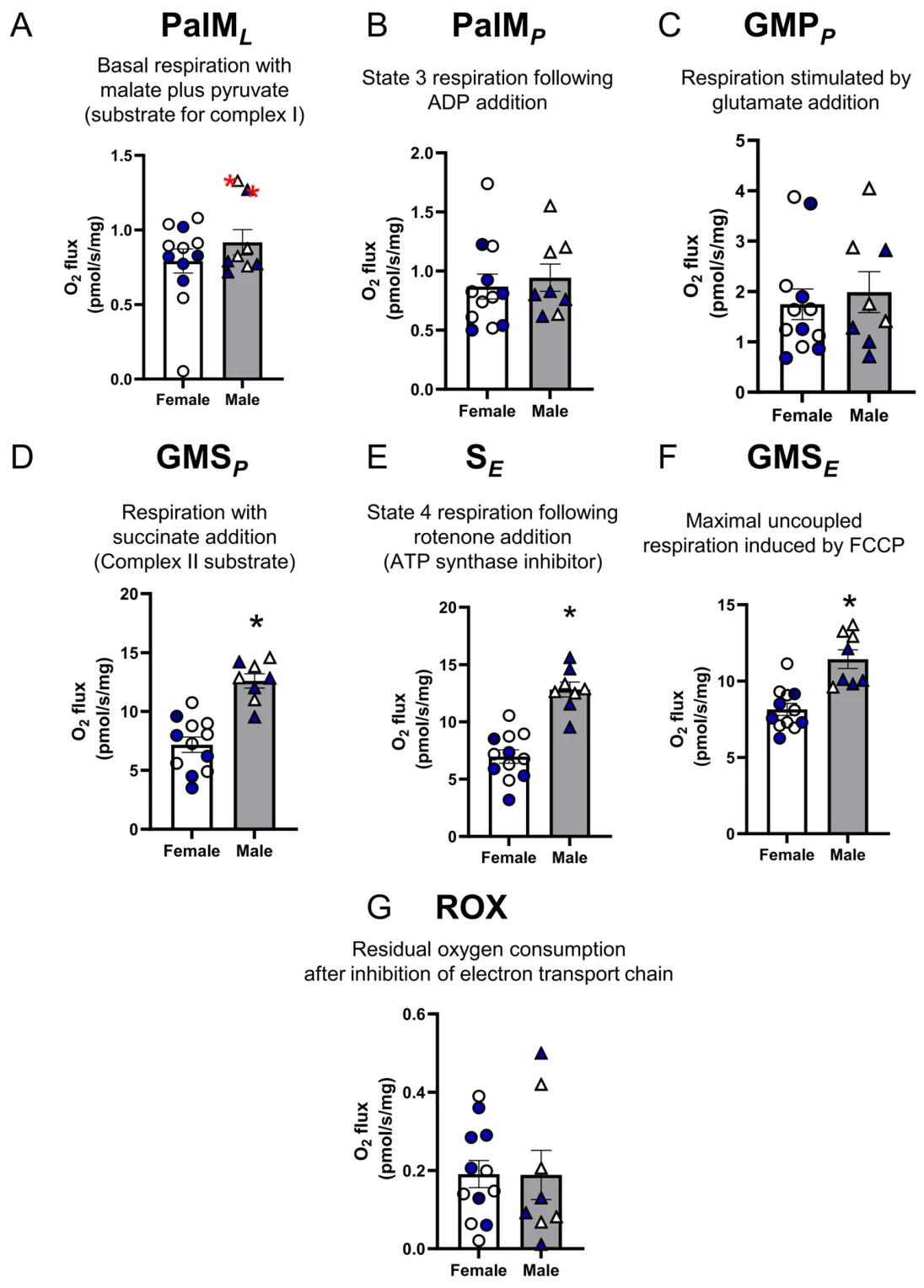
Placental mitochondrial respiratory capacity assessed using the Lipid SUIT protocol in female and male placentas. Mitochondrial respiratory function in term placental villous tissue was measured using the lipid substrate–uncoupler–inhibitor titration (Lipid SUIT) protocol and expressed as oxygen flux normalized to placental wet weight. β-Oxidation-linked LEAK respiration supported by malate and palmitoyl-carnitine (PalM*_L_*) (**A**); ADP-stimulated β-oxidation-supported oxidative phosphorylation with malate and palmitoyl-carnitine (PalM*_P_*) (**B**); glutamate-supported complex I oxidative phosphorylation (GMP*_P_*) (**C**); maximal oxidative phosphorylation capacity following succinate addition, reflecting convergent complex I and II electron flow (GMS*_P_*) (**D**); complex II-supported respiration following rotenone inhibition of complex I (S*_E_*) (**E**); maximal uncoupled respiratory capacity induced by FCCP (GMS*_E_*) (**F**); and residual oxygen consumption following inhibition of the electron transport chain with antimycin A (ROX) (**G**). Data are presented as means ± SEMs. * *p* < 0.05 versus female placentas. Statistical analyses were performed using an unpaired Student’s *t*-test or Mann–Whitney test. Two outliers (male samples) were identified in the PalM_L_ analyses using Grubbs’ test (α = 0.05); however, no outliers were excluded from the analyses. No outliers were identified for the PalM_P_, GMP_P_, GMS_P_, S_E_, GMS_E_, or ROX analyses. Final sample sizes were *n* = 12 female placentas and *n* = 8 male placentas depending on the analysis. Data shown in panels (**B**,**D**–**G**) were normally distributed, and statistical differences were evaluated using an unpaired Student’s *t*-test. Data shown in panels (**A**,**C**) were not normally distributed and were analyzed using the Mann–Whitney test. Abbreviations: PalM*_L_*, palmitoyl-carnitine/malate LEAK respiration; PalMP, palmitoyl-carnitine/malate oxidative phosphorylation; GMP*_P_*, glutamate-supported oxidative phosphorylation; GMS*_P_*, glutamate/malate/succinate oxidative phosphorylation; S*_E_*, succinate-supported respiration; GMS*_E_*, maximal uncoupled electron transport system capacity; FCCP, carbonyl cyanide-p-trifluoromethoxy phenylhydrazone; ROX, residual oxygen consumption. To illustrate the distribution of delivery modes within each sex, samples from vaginal deliveries are highlighted in blue, whereas white symbols indicate placentas delivered via cesarean section. The red asterisk indicates an outlier, included in the statistical analysis. All respiratory flux values were corrected for residual oxygen consumption (ROX) by subtracting ROX from each respiratory state before normalization to the tissue wet weight and statistical analysis.

In the Lipid SUIT protocol, no significant sex differences were observed in fatty acid oxidation (FAO)-linked LEAK respiration supported by palmitoyl–carnitine and malate (PalM*_L_*; *p* = 0.99; Figure 7A). Similarly, ADP-stimulated respiration supported by fatty acid substrates (PalM*_P_*, *p* = 0.79) and glutamate-supported complex I oxidative phosphorylation (GMP*_P_*, *p* = 0.57) did not differ between female and male placentas (Figure 7B,C).

In contrast, the maximal oxidative phosphorylation capacity following succinate addition (GMS*_P_*), representing convergent electron flow through complexes I and II, was 79% higher in male placentas (*p* = 0.0001; Figure 7D). Following the inhibition of complex I, succinate-supported complex II respiration (S*_E_*) was also elevated in male placentas by 79% (*p* = 0.0001; Figure 7E). Similarly, FCCP-induced maximal uncoupled respiration (GMS*_E_*), reflecting the maximal electron transport system capacity, was 35% higher in male placentas relative to female placentas (*p* = 0.002; Figure 7F). Residual oxygen consumption (ROX) following inhibition of the electron transport chain was not significantly different between groups (*p* = 0.97; Figure 7G), indicating comparable non-mitochondrial oxygen consumption. Collectively, these findings demonstrate enhanced maximal mitochondrial oxidative capacity in male placentas during fatty acid-supported respiration.

No significant sex differences were observed in the FCRs for β-oxidation-linked LEAK respiration supported by palmitoyl-carnitine and malate (PalM_L_; *p* = 0.36), ADP-stimulated β-oxidation-supported oxidative phosphorylation (PalM_P_; *p* = 0.14), or glutamate-supported complex I respiration (GMP_P_; *p* = 0.33) (Supplemental Figure S5A–C). In contrast, male placentas exhibited significantly higher FCRs for maximal oxidative phosphorylation following succinate addition (GMS_P_; *p* = 0.01) and for succinate-supported complex II respiration following rotenone inhibition (S_E_; *p* = 0.02) compared with female placentas (Supplemental Figure S5D,E). These findings indicate that, in addition to higher absolute respiratory capacity, male placental mitochondria exhibit a greater relative contribution of convergent complex I/II oxidative phosphorylation and complex II-supported respiration to maximal electron transport system capacity during fatty acid-supported respiration.

### 3.7. No Sex Differences in Placental Mitochondrial Electron Transport Chain Complex Protein Expression

**Figure 8:**
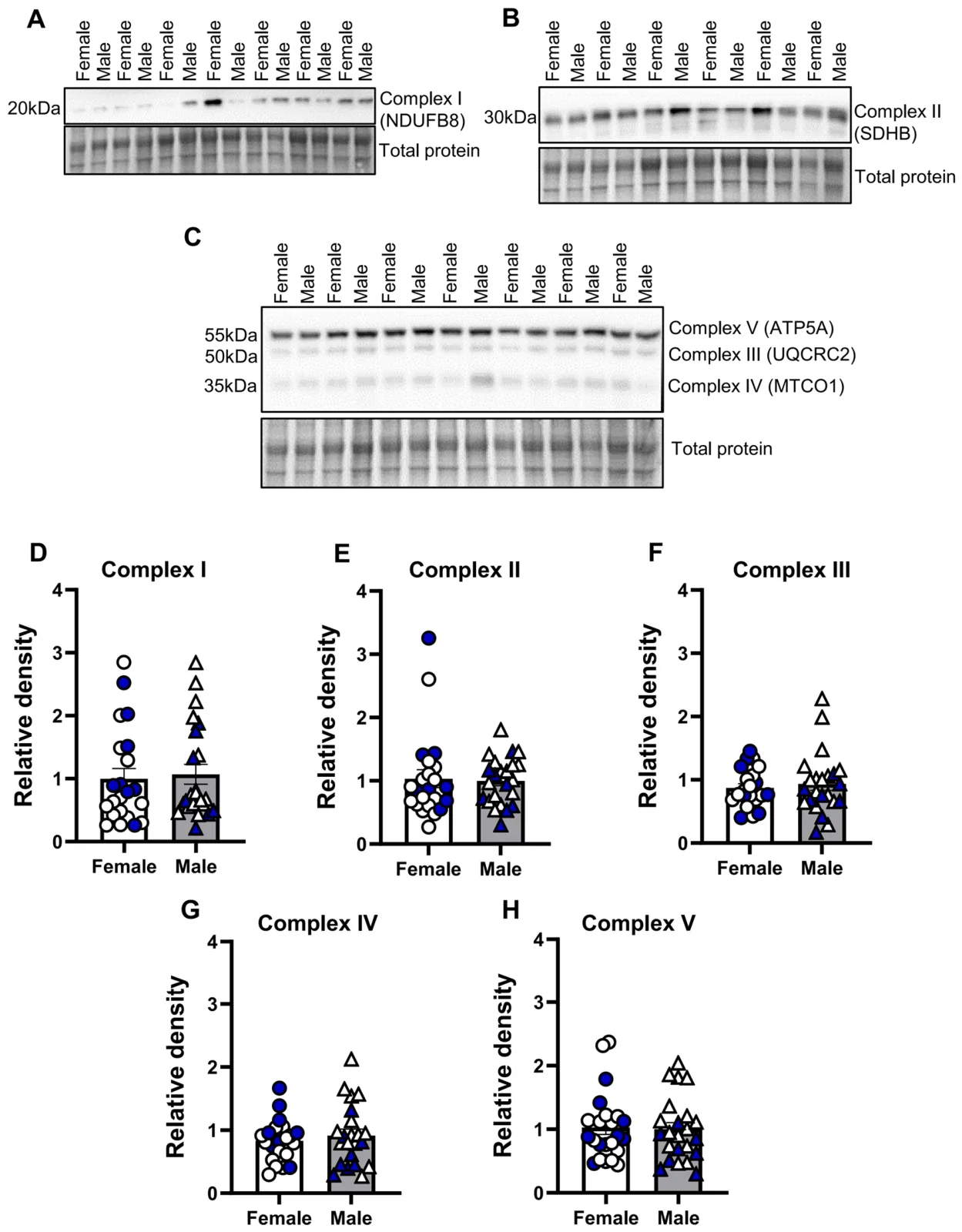
Protein expression of mitochondrial electron transport chain complexes in female and male placentas. Representative Western blots and quantification of Western blot analyses for mitochondrial electron transport chain complex I (NDUFB8) (**A**), complex II (SDHB) (**B**), complex III (UQCRC2) (**C**), complex IV (MTCO1) (**C**), and complex V (ATP5A) (**C**) protein expression in homogenates from female and male term placentas. (**D**–**H**) Histograms summarize densitometric analyses of Western blots. For each protein target, the mean density of female sample bands was assigned an arbitrary value of 1.0. Data are presented as means ± SEMs. Statistical analyses were performed using an unpaired Student’s *t*-test or Mann–Whitney test. One outlier (female sample) was removed from complex I analyses, two outliers (female samples) were removed from complex III analyses, and three outliers were removed from complex IV analyses (two female and one male sample) using Grubbs’ test (α = 0.05). No outliers were identified for complex II or complex V analyses. Final sample sizes were *n* = 21–23 female placentas and *n* = 21–23 male placentas depending on the analysis. Two outliers were removed from complex I analyses (female sample), two outliers were removed from complex II analyses (female sample), and one outlier was removed from complex III analyses (male sample) using the ROUT method (α = 0.01). Data shown in panel (**G**) were normally distributed, and the statistical difference was evaluated using an unpaired Student’s *t*-test. Data shown in panels (**D**–**F**,**H**) were not normally distributed and were analyzed using the Mann–Whitney test. Abbreviations: NDUFB8, NADH: ubiquinone oxidoreductase subunit B8; SDHB, succinate dehydrogenase complex iron sulfur subunit B; UQCRC2, ubiquinol–cytochrome c reductase core protein 2; MTCO1, mitochondrially encoded cytochrome c oxidase I; ATP5A, ATP synthase F1 subunit alpha. To illustrate the distribution of delivery modes within each sex, samples from vaginal deliveries are highlighted in blue, whereas white symbols indicate placentas delivered via cesarean section. All respiratory flux values were corrected for residual oxygen consumption (ROX) by subtracting ROX from each respiratory state before normalization to the tissue wet weight and statistical analysis. The molecular weight marker (kDa) is indicated on the left of each representative immunoblot. The expected molecular weights of complex I (NDUFB8), complex II (SDHB), complex III (UQCRC2), complex IV (MTCO1), and complex V (ATP5A) are 20, 30, 50, 35, and 55 kDa, respectively. Full-length, uncropped Western blot images including molecular weight markers are provided in the Supplemental Material.

Protein expression of mitochondrial electron transport chain (ETC) complexes I–V was assessed to determine whether sex-specific differences in placental mitochondrial respiration were associated with an altered abundance of oxidative phosphorylation machinery. These complexes are essential for mitochondrial ATP production and cellular energy metabolism, and changes in their expression may contribute to altered respiratory capacity. No significant sex differences were observed in the protein expression of complex I (NDUFB8, *p* = 0.39; Figure 8A,D), complex II (SDHB, *p* = 0.19; Figure 8B,E), complex III (UQCRC2, *p* = 0.88; Figure 8C,F), complex IV (MTCO1, *p* = 0.51; Figure 8C,G), or complex V (ATP5A, *p* = 0.88; Figure 8C,H) between female and male placentas. These findings indicate that the greater mitochondrial respiratory capacity observed in male placentas was not accompanied by differences in the abundance of ETC complex proteins, suggesting that functional regulation rather than the mitochondrial content may underlie the observed sex-specific differences in placental mitochondrial respiration.

### 3.8. Assessment of the Influence of Delivery Mode on Placental Signaling

Because labor and the mode of delivery are known to influence placental signaling by activating inflammatory, stress-related, and metabolic signaling pathways [59], we evaluated whether placental signaling protein expression differed between vaginal and cesarean deliveries within each fetal sex. This analysis was performed to determine whether the observed sex-specific differences in placental signaling could be confounded by the delivery mode. In female placentas, total AMPK protein expression differed significantly between delivery modes (*p* = 0.03), whereas no significant differences were observed for any of the other signaling proteins (Supplemental Table S6 and Supplemental Figure S6A–D). In male placentas, none of the signaling proteins differed significantly between cesarean and vaginal deliveries (Supplemental Table S6). These findings indicate that the delivery mode had minimal influence on the placental signaling pathways examined and is therefore unlikely to account for the observed sex-specific differences in placental signaling.

### 3.9. Maternal BMI Is Not Associated with Placental Metabolic Signaling, Amino Acid Transport, or Mitochondrial Protein Expression

To determine whether the maternal BMI contributed to the variability observed in placental signaling, amino acid transport, and mitochondrial protein expression, correlation analyses were performed separately for female and male placentas. Maternal BMI was not significantly associated with most measured parameters. For mTOR signaling (Supplemental Figures S7A–D and S8A–D), no significant correlations were observed between maternal BMI and total S6RP (female: r = 0.15, *p* = 0.49; male: r = –0.02, *p* = 0.94), phosphorylated S6RP (female: r = –0.06, *p* = 0.80; male: r = 0.10, *p* = 0.67), total 4E-BP1 (female: r = –0.11, *p* = 0.62; male: r = –0.16, *p* = 0.49), or phosphorylated 4E-BP1 (female: r = –0.53, *p* = 0.094; male: r = 0.32, *p* = 0.13).

For AKT signaling (Supplemental Figure S9A–F), total AKT showed no significant association with maternal BMI (female: r = 0.23, *p* = 0.30; male: r = 0.22, *p* = 0.32), nor did phosphorylated AKT (Ser473) (female: r = –0.01, *p* = 0.62; male: r = 0.10, *p* = 0.64). In contrast, phosphorylated AKT (Thr308) was positively correlated with the maternal BMI in male placentas (r = 0.55, *p* = 0.007) but not female placentas (r = –0.14, *p* = 0.51). Total AMPK (Supplemental Figure S10A–B) showed a trend toward a positive correlation with the maternal BMI in male placentas (r = 0.39, *p* = 0.06) but not female placentas (r = –0.30, *p* = 0.17), while phosphorylated AMPK was not significantly associated with the maternal BMI in either females (r = –0.18, *p* = 0.41) or males (r = –0.37, *p* = 0.09).

Likewise, OGT (female: r = –0.23, *p* = 0.29; male: r = –0.07, *p* = 0.73), total ERK1/2 (female: r = 0.05, *p* = 0.81; male: r = 0.06, *p* = 0.80), and phosphorylated ERK1/2 (female: r = –0.19, *p* = 0.40; male: r = 0.40, *p* = 0.06) were not significantly correlated with the maternal BMI (Supplemental Figure S11A–F). Similarly (Supplemental Figure S12A–H), no significant correlations were observed between the maternal BMI and System A amino acid transport activity (female: r = –0.53, *p* = 0.14; male: r = 0.26, *p* = 0.46), SNAT1 (female: r = 0.14, *p* = 0.69; male: r = –0.04, *p* = 0.92), SNAT2 (female: r = –0.54, *p* = 0.11; male: r = 0.003, *p* = 0.99), or SNAT4 (female: r = 0.25, *p* = 0.49; male: r = 0.29, *p* = 0.42).

Finally (Supplemental Figure S13A–F), the maternal BMI was not associated with mitochondrial electron transport chain complex I (female: r = 0.18, *p* = 0.41; male: r = 0.03, *p* = 0.89), complex II (female: r = –0.14, *p* = 0.51; male: r = –0.12, *p* = 0.59), complex III (female: r = –0.31, *p* = 0.18; male: r = –0.03, *p* = 0.91), or complex IV (female: r = –0.35, *p* = 0.12; male: r = 0.01, *p* = 0.96). The only significant association observed was a negative correlation (Supplemental Figure S14A–D) between the maternal BMI and mitochondrial complex V protein expression in female placentas (r = –0.51, *p* = 0.012), whereas no association was present in male placentas (r = –0.03, *p* = 0.88). Collectively, these findings indicate that the maternal BMI is unlikely to account for the variability observed in placental signaling, amino acid transport, or mitochondrial protein expression.

## 4. Discussion

Sexual dimorphism in placental function and fetal growth is increasingly recognized as an important determinant of pregnancy outcomes and developmental programming. Male fetuses typically exhibit faster growth trajectories and greater birth weights than female fetuses, suggesting sex-specific differences in placental nutrient sensing and metabolic regulation. The present study demonstrates that male term placentas exhibit increased mTORC1 signaling activity, greater System A amino acid transport capacity, and enhanced mitochondrial respiratory capacity as compared to female placentas. Specifically, male placentas showed increased phosphorylation of S6RP, reduced 4EBP1 expression, higher MVM System A amino acid transport activity, and elevated maximal oxidative phosphorylation and electron transport system capacity. These findings provide new evidence for the sex-specific regulation of placental signaling and metabolism and suggest that enhanced placental anabolic and bioenergetic function in male placentas may contribute to the accelerated growth trajectory of male fetuses. The significantly higher birth weight-to-placental weight ratio in male pregnancies suggests that male placentas support greater fetal growth despite a similar placental mass, consistent with increased placental functional efficiency. Nevertheless, because this was a cross-sectional study, our findings demonstrate associations among placental signaling, nutrient transport, mitochondrial function, and fetal growth, rather than establishing a causal relationship.

Using S6RP phosphorylation as a functional readout for mTORC1 activity [60], we found evidence for higher mTORC1 activity in male as compared to female placentas. This conclusion is further supported by the lower total 4EBP1 protein expression in male placentas. 4EBP1 is not a kinase but a binding protein that prevents cap-dependent translation initiation by binding eukaryotic translation initiation factor 4E (eIF4E), and the phosphorylation of 4EBP1 blocks this binding [61,62]. Thus, decreased total 4EBP1 expression is associated with activation of the pathway because it will result in lower binding of eIF4E, thereby promoting protein translation. The previous report of increased *mTOR* mRNA levels in male as compared to female placenta [33] is in general agreement with our findings. Placental mTOR signaling is regulated by an array of upstream signals, including insulin/IGF-1 signaling, AMPK, and the availability of amino acids, glucose, folate, fatty acids, and oxygen. However, changes in AMPK or insulin/IGF-1 signaling are, given the lack of sexual dimorphism in the activity of these signaling pathway in the current study, unlikely to explain the mTOR activation in male placentas.

The absence of sex differences in upstream AKT and AMPK signaling despite enhanced downstream mTORC1 activity in male placentas suggests that alternative regulatory mechanisms may contribute to mTORC1 activation. This apparent dissociation between upstream signaling pathways and downstream mTORC1 activation may reflect the complex and multifactorial regulation of mTORC1 activity in the placenta [63]. In addition to PI3K/AKT and AMPK signaling, mTORC1 is highly responsive to intracellular amino acid availability [64], nutrient sensing pathways [65], oxygen tension [66], mitochondrial metabolism [67], and Rag GTPase-mediated signaling [68]. Accordingly, enhanced downstream mTORC1 activation in male placentas may occur independently of detectable differences in basal AKT or AMPK phosphorylation. Moreover, AKT and AMPK signaling are highly dynamic and often change only under specific metabolic or pathological stimuli such as fetal growth restriction [69], gestational diabetes [69], maternal overweight [70], or maternal immune activation [71]. In contrast, healthy term placentas frequently exhibit stable AKT and AMPK phosphorylation despite alterations in downstream mTORC1 targets such as rpS6 [69], suggesting that single timepoint measurements at delivery may not capture transient or earlier upstream signaling events contributing to downstream pathway activation. Additionally, compensatory regulatory mechanisms may contribute to the preservation of similar upstream signaling between sexes despite divergent downstream responses. For example, the inverse sex-dependent expression of AKT regulators such as PTEN and Klotho has been shown to be associated to comparable AKT activity between males and females in the brain [72], while translational compensation mechanisms can mask sex differences even when transcript expression differs, as reported for AMPKα1 expression in avian liver models [73]. Consistent with these observations, placentas from our cohort of uncomplicated term pregnancies demonstrated sex-specific differences in downstream mTORC1 signaling without corresponding alterations in upstream AKT or AMPK pathways. The higher total AMPK protein expression demonstrated in female placentas delivered via cesarean section may reflect the differential regulation of AMPK abundance by labor-associated physiological stress [59]. Vaginal delivery is associated with an increased metabolic demand, intermittent hypoxia, and endocrine changes that can influence placental signaling pathways. AMPK is a central metabolic sensor that integrates these signals and has been implicated in regulating uteroplacental blood flow, nutrient transport, inflammatory responses, uterine receptivity, and myometrial contractility [74,75,76,77,78,79,80,81]. In fetal membranes, AMPK activity has been reported to decrease during spontaneous term labor, whereas the pharmacological activation of AMPK suppresses pro-inflammatory and pro-labor mediators, supporting a role for AMPK in the regulation of labor [75]. However, because AMPK phosphorylation was unchanged, these data suggest that the mode of delivery affected AMPK protein abundance rather than its activation. Moreover, direct evidence for the sex-specific regulation of placental AMPK based on the mode of delivery is limited.

The higher total AMPK protein expression observed in female placentas delivered via cesarean section may reflect the differential regulation of AMPK abundance in response to labor-associated physiological stress [65]. Vaginal delivery is accompanied by an increased metabolic demand, intermittent hypoxia, inflammatory activation, and endocrine changes that influence placental and uterine signaling pathways [80,81,82,83,84,85,86,87]. AMPK is a central metabolic sensor that integrates these signals and has been implicated in regulating the uteroplacental blood flow, inflammatory responses, uterine receptivity, and myometrial contractility. In fetal membranes, AMPK activity has been reported to decrease during spontaneous term labor, whereas the pharmacological activation of AMPK suppresses pro-inflammatory and pro-labor mediators, supporting a role for AMPK in the regulation of labor. However, because AMPK phosphorylation was unchanged in the present study, our findings suggest that the mode of delivery influenced AMPK protein abundance rather than its activation. Moreover, direct evidence for the sex-specific regulation of placental AMPK based on the mode of delivery is limited. Therefore, the observed increase in total AMPK protein expression in female placentas delivered via cesarean section should be interpreted cautiously and requires confirmation in larger cohorts to determine whether it represents a true sex-specific adaptation to labor or a chance finding.

In the current study, we found no sex difference in placental OGT protein expression at term, which is consistent with our recent findings in human term placentas [47]. In contrast, a previous study reported lower OGT protein expression in male compared with female placentas at term [46]; however, that study included a relatively small sample size. It is also possible that sex-specific differences in placental OGT expression vary across gestation. Supporting this concept, we previously demonstrated that OGT protein expression is higher in female than male placentas in the first trimester [47]. Although mTOR signaling has been reported to regulate placental OGT protein expression, the absence of sex differences in total OGT protein abundance despite enhanced downstream mTORC1 signaling in male placentas suggests that OGT protein expression is also regulated by other signaling pathways.

Placental amino acid transport capacity has been reported to be decreased in infants born growth restricted and increased in LGA infants [32,82,83,84,85], consistent with the possibility that placental amino acid transport activity is mechanistically linked to fetal growth. Indeed, the placenta-specific knock-down of SNAT2, an isoform of the System A transport system, caused fetal growth restriction in mice [86]. We also reported that the placenta-specific overexpression of LAT1, an isoform of the System L transporter, resulted in fetal overgrowth [87], confirming a cause-and-effect relationship between placental amino acid transport activity and fetal growth. Emerging evidence further suggests that placental System A transport exhibits sex-specific regulation, particularly under conditions of metabolic or inflammatory stress. Experimental studies have demonstrated the male-specific upregulation of *Slc38a1*, *Slc38a2*, and *Slc38a4* expression during maternal intermittent fasting [88], despite reductions in overall System A transport activity, suggesting compensatory but potentially insufficient adaptive responses in male placentas. Similarly, maternal high-fat-diet exposure has been associated with selective increases in System A expression in male placentas [89], whereas inflammatory and preeclampsia models indicate greater male vulnerability to impaired amino acid availability despite minimal changes in transporter expression [83].

The higher System A amino acid transport activity in MVM vesicles isolated from male placentas in the current study may contribute to the increased in utero fetal growth rate of boys. Recently it was reported that boys have a significantly lower adiposity at birth than girls [90], and it is possible that the higher amino acid transporter capacity in term male placenta contributes to this sex difference in body composition. mTOR signaling positively regulates the trafficking and activity of SNAT2, a major isoform of the System A amino acid transporter [24], and increased System A transport activity in male placentas could therefore have reflected the greater plasma membrane abundance of SNAT2. However, MVM SNAT2 protein expression did not differ between male and female placentas. Similarly, the MVM protein expression of SNAT 1 and 4 was not different between male and female placentas, suggesting that mechanisms other than changes in the total abundance of System A amino acid transporter isoforms may underlie the observed sexual dimorphism in System A transport activity. These mechanisms may instead involve the post-translational regulation of transporter activity or differences in transporter kinetics. This interpretation is supported by experimental studies demonstrating that the activation of protein kinase C increases amino acid transporter activity through changes in transport kinetics (Vmax) without alterations in transporter expression or membrane insertion [91], consistent with the possibility that enhanced System A activity in male placentas may reflect the functional activation of existing transporters rather than increased transporter abundance.

Several studies have suggested an association between the expression of proteins in the electron transport chain of mitochondrial respiration and fetal growth [25,92]. For example, placental complex I-stimulated oxygen consumption was reported to be decreased in FGR [92]. Our recent study shows that the protein expression of ETS complexes is significantly reduced in FGR placentas [25]. In the current study, we found that the respiratory capacity of placental mitochondria at term is higher in males as compared to females. Specifically, we observed a greater oxidative phosphorylation capacity, enhanced complex II-linked respiration, and increased maximal ETS capacity in male as compared to female placentas. In particular, the higher convergent complex I and II-supported respiration in male placentas suggests enhanced mitochondrial function during conditions of increased energetic demand. Furthermore, the increased succinate-driven respiration following complex I inhibition suggests that male placentas rely more heavily on complex II-supported electron transport, consistent with previous reports of greater mitochondrial respiratory capacity in male placentas under metabolic stress conditions. Additionally, the greater FCCP-induced uncoupled respiration in male placentas indicates higher maximal electron transport system capacity and supports a high-capacity oxidative phenotype. Collectively, these findings suggest that male placentas adopt a high-flux oxidative strategy characterized by an enhanced maximal respiratory capacity and greater reliance on complex II-linked respiration, potentially representing an adaptive mechanism to support the increased anabolic and energetic demands associated with male fetal growth. Importantly, to our knowledge, this is the first study to identify sex-specific increases in convergent complex I and II oxidative phosphorylation capacity, succinate-supported complex II respiration, and maximal ETS capacity in human placental tissue using a Carbohydrate and Lipid SUIT protocol.

Despite the higher mitochondrial respiratory capacity observed in male placentas, we did not detect any sex differences in mitochondrial ETS complex protein expression, suggesting that functional regulation rather than differences in the mitochondrial content or ETS abundance underlies the observed respiratory phenotype. In other studies, the inhibition of mTOR signaling decreases the gene expression of the mitochondrial transcriptional regulators PGC-1α in skeletal muscle tissues [93], and the activation of mTORC1 increases mitochondrial DNA expression, mitochondrial density, and ATP production in pancreatic beta cells [94]. Moreover, in MCF7 cells, mTORC1 controls mitochondrial activity and biogenesis mediated by 4E-BP1-dependent translational regulation [95]. The activation of mTORC1 signaling in primary human trophoblast cells promotes mitochondrial respiration mediated, at least in part, by increasing mitochondrial biogenesis [25]. Thus, higher mitochondrial respiration in male placentas may be linked to the higher mTORC1 activity in male placentas. However, our data do not suggest that differences in ETS protein expression and/or the mitochondrial number underpin the sexual dimorphism in placental mitochondrial respiration. Instead, the increased mitochondrial respiration in male placentas may reflect enhanced mitochondrial functional activity, altered substrate utilization, greater electron transport efficiency, or differences in mitochondrial regulatory pathways independent of ETS complex abundance. It has been reported that placental complex II related-respiration is reduced in preeclampsia, whereas the protein expression of complex II is not changed and placental mitochondrial reactive oxygen species (ROS) are reduced [96]. ROS may be the key to understanding the difference in complex II-related respiration between male and female placentas, but studies on the sex difference in placental ROS are limited. Further studies are needed to determine if mTORC1 regulates mitochondrial respiration via mechanisms other than the effects of mitochondrial biogenesis or if the higher mitochondrial respiration in male placentas is mediated by other signaling pathways.

This study has several limitations. First, the cross-sectional study design precludes conclusions regarding causality; therefore, the observed associations among placental signaling, nutrient transport, mitochondrial function, and fetal growth should not be interpreted as causal. Second, although the mitochondrial respiration analyses were performed in an expanded cohort, the sample size remained modest, and these findings should be confirmed in larger studies. Third, the mitochondrial content was inferred from electron transport chain complex protein expression rather than direct measures such as citrate synthase activity or the mitochondrial DNA copy number. To address this limitation, we calculated flux control ratios, which supported intrinsic differences in mitochondrial respiratory function between male and female placentas. Finally, the maternal age tended to be higher in pregnancies carrying female fetuses, although this difference was not statistically significant. While maternal age may influence placental biology, the modest difference observed between groups is unlikely to account for the marked sex-specific differences in placental signaling, amino acid transport, and mitochondrial respiratory function observed in this study.

Emerging evidence suggests that placental amino acid transport and mitochondrial function are critical in determining fetal growth and development [86,97]. Although sex differences in fetal growth and pregnancy outcomes have been observed for centuries [1] and there is ample evidence that placental gene expression varies with fetal sex [18,19,20,21], whether the functions of the male and female placenta differ remains to be fully established. Thus, insights into sexual dimorphism in placental function in normal pregnancy may help us better understand sex differences in adverse pregnancy outcomes. We have previously reported that placental signaling pathways such as insulin/IGF-1 and mTOR are key regulators of placental amino acid transport activity, which is positively associated with infant birthweight. We propose that the higher placental mTOR signaling activity, System A amino acid transport capacity, and mitochondrial respiratory capacity in males reported in this study may contribute to faster in utero growth in boys.

## Figures and Tables

**Table 1:** Selected clinical characteristics for study subjects.

	Female Fetuses (*n* = 23)	Male Fetuses (*n* = 23)	*p* Value
Maternal age (years)	31.87 ± 1.02 [23–40]	29.39 ± 1.12 [18–37]	0.11
BMI (kg/m^2^)	22.4 ± 0.41 [18.90–25.78]	23.0 ± 0.43 [19.42–28.31]	0.34
Number of overweight women (*n*)	2	4	0.67
Gestational age (weeks)	39.19 ± 0.21 [37.29–41.29]	39.37 ± 0.15 [38.29–41.29]	0.47
Birth weight (g)	3209 ± 57.7 [2718–3800]	3401 ± 65.7 [2955–4094]	0.03
Birth weight z-score	−0.14 ± 0.12 [−1.20–1.03]	−0.09 ± 0.10 [−0.91–0.70]	0.78
Placental weight (g)	627 ± 19.6 [437.00–873.43]	608 ± 23.0 [388.60–849.00]	0.54
Birthweight-to-placental weight ratio	5.17 ± 0.13 [3.77–6.62]	5.77 ± 0.23 [3.76–7.94]	0.03
Delivery mode (C/V)	15/8	15/8	1.00
**Race** White Black Asian Unknown	18 (78%) 4 (18%) - 1 (4%)	18 (78%) 1 (4%) 3 (14%) 1 (4%)	1.00
Ethnicity Hispanic Non-Hispanic	19 (83%) 4 (17%)	19 (83%) 4 (17%)	1.00

Data are presented as means ± SEMs [minimum–maximum]. Abbreviations: *n*, numbers; C, caesarean section; V, vaginal. The statistical significance of differences between female and male groups was assessed using unpaired Student’s *t*-tests except for the Number of women with overweight, Delivery mode, and Ethnicity, for which Fisher’s test was used.

## Data Availability

The authors confirm that the data supporting the findings of the present study are available within the article, its Supplementary Materials, and from the corresponding authors upon reasonable request.

## Supplementary Materials

The following supporting information can be downloaded at: https://www.mdpi.com/article/10.3390/antiox15080977/s1, Supplemental Table S1: Primary and secondary antibodies used for Western blot analyses; Supplemental Table S2: Sequential antibody probing following membrane stripping in Western blot analyses; Supplemental Table S3: Selected clinical characteristics of subjects included in MVM amino acid transport experiments; Supplemental Table S4: Selected clinical characteristics for subjects included in placental oxygen consumption rate experiments; Supplemental Table S5: Outliers identified by Grubbs’ test in placental signaling Western blot analyses; Supplemental Table S6: Assessment of the effect of delivery mode (vaginal versus cesarean delivery) on placental signaling pathway readouts; Supplemental Figure S1: Ratios of phosphorylated 4E-BP1 and S6RP to total protein expression in female and male placentas; Supplemental Figure S2: Ratios of phosphorylated AMPK, AKT, and ERK1/2 to total AMPK, AKT, and ERK1/2 protein expression in female and male placentas; Supplemental Figure S3: Time course of Na+-dependent MeAIB uptake in placental MVM vesicles; Supplemental Figure S4: Flux control ratios of placental mitochondrial respiratory states assessed using the Carb SUIT protocol in female and male placentas; Supplemental Figure S5: Flux control ratios (FCRs) of mitochondrial respiratory states assessed using the Lipid SUIT protocol in female and male placentas; Supplemental Figure S6: Effect of mode of delivery on AMPK protein expression and phosphorylated AMPK/total AMPK ratio in female and male placentas; Supplemental Figure S7: Correlation between maternal BMI and S6RP protein expression/phosphorylation in female and male placentas; Supplemental Figure S8: Correlation between maternal BMI and 4E-BP1 protein expression/phosphorylation in female and male placentas; Supplemental Figure S9: Correlation between maternal BMI and AKT protein expression/phosphorylation in female and male placentas; Supplemental Figure S10: Correlation between maternal BMI and AMPK protein expression/phosphorylation in female and male placentas; Supplemental Figure S11: Correlation between maternal BMI and OGT and ERK1/2 protein expression-phsophorylation in female and male placentas; Supplemental Figure S12: Correlation between maternal BMI and System A amino acid transport activity and SNAT protein expression in female and male placentas; Supplemental Figure S13: Correlation between maternal BMI and mitochondrial electron transport chain complex I–III protein expression in female and male placentas; Supplemental Figure S14: Correlation between maternal BMI and mitochondrial electron transport chain complex IV and V protein expression in female and male placentas.

## References

[1] Clarke J. (1788). Observations on Some Causes of the Excess of the Mortality of Males above That of Females. Lond. Med. J..

[2] Ray P.F., Conaghan J., Winston R.M., Handyside A.H. (1995). Increased number of cells and metabolic activity in male human preimplantation embryos following in vitro fertilization. J. Reprod. Fertil..

[3] Ménézo Y.J., Chouteau J., Torelló J., Girard A., Veiga A. (1999). Birth weight and sex ratio after transfer at the blastocyst stage in humans. Fertil. Steril..

[4] Broere-Brown Z.A., Baan E., Schalekamp-Timmermans S., Verburg B.O., Jaddoe V.W., Steegers E.A. (2016). Sex-specific differences in fetal and infant growth patterns: A prospective population-based cohort study. Biol. Sex Differ..

[5] Galjaard S., Ameye L., Lees C.C., Pexsters A., Bourne T., Timmerman D., Devlieger R. (2019). Sex differences in fetal growth and immediate birth outcomes in a low-risk Caucasian population. Biol. Sex Differ..

[6] Bekedam D.J., Engelsbel S., Mol B.W., Buitendijk S.E., van der Pal-de Bruin K.M. (2002). Male predominance in fetal distress during labor. Am. J. Obstet. Gynecol..

[7] Matthews T.J., MacDorman M.F., Thoma M.E. (2015). Infant Mortality Statistics From the 2013 Period Linked Birth/Infant Death Data Set. Natl. Vital. Stat. Rep..

[8] Wong C., Schreiber V., Crawford K., Kumar S. (2023). Male infants are at higher risk of neonatal mortality and severe morbidity. Aust. N. Z. J. Obstet. Gynaecol..

[9] Peelen M.J., Kazemier B.M., Ravelli A.C., De Groot C.J., Van Der Post J.A., Mol B.W., Hajenius P.J., Kok M. (2016). Impact of fetal gender on the risk of preterm birth, a national cohort study. Acta Obstet. Gynecol. Scand..

[10] Melamed N., Yogev Y., Glezerman M. (2010). Fetal gender and pregnancy outcome. J. Matern.-Fetal Neonatal Med..

[11] Elsmén E., Källén K., Marsál K., Hellström-Westas L. (2006). Fetal gender and gestational-age-related incidence of pre-eclampsia. Acta Obstet. Gynecol. Scand..

[12] Di Renzo G.C., Rosati A., Sarti R.D., Cruciani L., Cutuli A.M. (2007). Does fetal sex affect pregnancy outcome?. Gend. Med..

[13] Cooperstock M., Campbell J. (1996). Excess males in preterm birth: Interactions with gestational age, race, and multiple birth. Obstet. Gynecol..

[14] Walker M.G., Fitzgerald B., Keating S., Ray J.G., Windrim R., Kingdom J.C. (2012). Sex-specific basis of severe placental dysfunction leading to extreme preterm delivery. Placenta.

[15] Broere-Brown Z.A., Adank M.C., Benschop L., Tielemans M., Muka T., Gonçalves R., Bramer W.M., Schoufour J.D., Voortman T., Steegers E.A.P. (2020). Fetal sex and maternal pregnancy outcomes: A systematic review and meta-analysis. Biol. Sex Differ..

[16] Schalekamp-Timmermans S., Arends L.R., Alsaker E., Chappell L., Hansson S., Harsem N.K., Jälmby M., Jeyabalan A., Laivuori H., Lawlor D.A. (2017). Fetal sex-specific differences in gestational age at delivery in pre-eclampsia: A meta-analysis. Int. J. Epidemiol..

[17] Aibar L., Puertas A., Valverde M., Carrillo M.P., Montoya F. (2012). Fetal sex and perinatal outcomes. J. Perinat. Med..

[18] Cox L.A., Li C., Glenn J.P., Lange K., Spradling K.D., Nathanielsz P.W., Jansson T. (2013). Expression of the placental transcriptome in maternal nutrient reduction in baboons is dependent on fetal sex. J. Nutr..

[19] Osei-Kumah A., Smith R., Jurisica I., Caniggia I., Clifton V.L. (2011). Sex-specific differences in placental global gene expression in pregnancies complicated by asthma. Placenta.

[20] Clifton V.L. (2010). Review: Sex and the human placenta: Mediating differential strategies of fetal growth and survival. Placenta.

[21] Barke T.L., Money K.M., Du L., Serezani A., Gannon M., Mirnics K., Aronoff D.M. (2019). Sex modifies placental gene expression in response to metabolic and inflammatory stress. Placenta.

[22] Shimada H., Powell T.L., Jansson T. (2024). Regulation of placental amino acid transport in health and disease. Acta Physiol..

[23] McIntyre K.R., Vincent K.M.M., Hayward C.E., Li X., Sibley C.P., Desforges M., Greenwood S.L., Dilworth M.R. (2020). Human placental uptake of glutamine and glutamate is reduced in fetal growth restriction. Sci. Rep..

[24] Rosario F.J., Dimasuay K.G., Kanai Y., Powell T.L., Jansson T. (2016). Regulation of amino acid transporter trafficking by mTORC1 in primary human trophoblast cells is mediated by the ubiquitin ligase Nedd4-2. Clin. Sci..

[25] Rosario F.J., Gupta M.B., Myatt L., Powell T.L., Glenn J.P., Cox L., Jansson T. (2019). Mechanistic Target of Rapamycin Complex 1 Promotes the Expression of Genes Encoding Electron Transport Chain Proteins and Stimulates Oxidative Phosphorylation in Primary Human Trophoblast Cells by Regulating Mitochondrial Biogenesis. Sci. Rep..

[26] Saxton R.A., Sabatini D.M. (2017). mTOR Signaling in Growth, Metabolism, and Disease. Cell.

[27] Wälchli M., Berneiser K., Mangia F., Imseng S., Craigie L.M., Stuttfeld E., Hall M.N., Maier T. (2021). Regulation of human mTOR complexes by DEPTOR. eLife.

[28] Dumolt J.H., Powell T.L., Jansson T. (2021). Placental Function and the Development of Fetal Overgrowth and Fetal Growth Restriction. Obstet. Gynecol. Clin. N. Am..

[29] Yung H.W., Calabrese S., Hynx D., Hemmings B.A., Cetin I., Charnock-Jones D.S., Burton G.J. (2008). Evidence of placental translation inhibition and endoplasmic reticulum stress in the etiology of human intrauterine growth restriction. Am. J. Pathol..

[30] Chen Y.Y., Rosario F.J., Shehab M.A., Powell T.L., Gupta M.B., Jansson T. (2015). Increased ubiquitination and reduced plasma membrane trafficking of placental amino acid transporter SNAT-2 in human IUGR. Clin. Sci..

[31] Sati L., Soygur B., Celik-Ozenci C. (2016). Expression of Mammalian Target of Rapamycin and Downstream Targets in Normal and Gestational Diabetic Human Term Placenta. Reprod. Sci..

[32] Jansson N., Rosario F.J., Gaccioli F., Lager S., Jones H.N., Roos S., Jansson T., Powell T.L. (2013). Activation of placental mTOR signaling and amino acid transporters in obese women giving birth to large babies. J. Clin. Endocrinol. Metab..

[33] Sedlmeier E.M., Meyer D.M., Stecher L., Sailer M., Daniel H., Hauner H., Bader B.L. (2021). Fetal sex modulates placental microRNA expression, potential microRNA-mRNA interactions, and levels of amino acid transporter expression and substrates: INFAT study subpopulation analysis of n-3 LCPUFA intervention during pregnancy and associations with offspring body composition. BMC Mol. Cell Biol..

[34] Gonzalez T.L., Sun T., Koeppel A.F., Lee B., Wang E.T., Farber C.R., Rich S.S., Sundheimer L.W., Buttle R.A., Chen Y.I. (2018). Sex differences in the late first trimester human placenta transcriptome. Biol. Sex Differ..

[35] Braun A.E., Muench K.L., Robinson B.G., Wang A., Palmer T.D., Winn V.D. (2021). Examining Sex Differences in the Human Placental Transcriptome During the First Fetal Androgen Peak. Reprod. Sci..

[36] Braun A.E., Mitchel O.R., Gonzalez T.L., Sun T., Flowers A.E., Pisarska M.D., Winn V.D. (2022). Sex at the interface: The origin and impact of sex differences in the developing human placenta. Biol. Sex Differ..

[37] Buckberry S., Bianco-Miotto T., Bent S.J., Dekker G.A., Roberts C.T. (2014). Integrative transcriptome meta-analysis reveals widespread sex-biased gene expression at the human fetal-maternal interface. Mol. Hum. Reprod..

[38] Neirijnck Y., Papaioannou M.D., Nef S. (2019). The Insulin/IGF System in Mammalian Sexual Development and Reproduction. Int. J. Mol. Sci..

[39] Aslam M., Ladilov Y. (2022). Emerging Role of cAMP/AMPK Signaling. Cells.

[40] Hardivillé S., Hart G.W. (2014). Nutrient regulation of signaling, transcription, and cell physiology by O-GlcNAcylation. Cell Metab..

[41] Hart B., Morgan E., Alejandro E.U. (2019). Nutrient sensor signaling pathways and cellular stress in fetal growth restriction. J. Mol. Endocrinol..

[42] Lubas W.A., Frank D.W., Krause M., Hanover J.A. (1997). O-Linked GlcNAc transferase is a conserved nucleocytoplasmic protein containing tetratricopeptide repeats. J. Biol. Chem..

[43] Gao Y., Wells L., Comer F.I., Parker G.J., Hart G.W. (2001). Dynamic O-glycosylation of nuclear and cytosolic proteins: Cloning and characterization of a neutral, cytosolic beta-N-acetylglucosaminidase from human brain. J. Biol. Chem..

[44] Lima V.V., Dela Justina V., Dos Passos R.R., Volpato G.T., Souto P.C.S., San Martin S., Giachini F.R. (2018). O-GlcNAc Modification During Pregnancy: Focus on Placental Environment. Front. Physiol..

[45] Groves J.A., Lee A., Yildirir G., Zachara N.E. (2013). Dynamic O-GlcNAcylation and its roles in the cellular stress response and homeostasis. Cell Stress Chaperones.

[46] Howerton C.L., Morgan C.P., Fischer D.B., Bale T.L. (2013). O-GlcNAc transferase (OGT) as a placental biomarker of maternal stress and reprogramming of CNS gene transcription in development. Proc. Natl. Acad. Sci. USA.

[47] Kelly A.C., Kramer A., Rosario F.J., Powell T.L., Jansson T. (2020). Inhibition of mechanistic target of rapamycin signaling decreases levels of O-GlcNAc transferase and increases serotonin release in the human placenta. Clin. Sci..

[48] Mackenzie B., Erickson J.D. (2004). Sodium-coupled neutral amino acid (System N/A) transporters of the SLC38 gene family. Pflug. Arch..

[49] Desforges M., Lacey H.A., Glazier J.D., Greenwood S.L., Mynett K.J., Speake P.F., Sibley C.P. (2006). The SNAT4 isoform of the system A amino acid transporter is expressed in human placenta. Am. J. Physiol. Cell Physiol..

[50] Cuffe J.S., Walton S.L., Singh R.R., Spiers J.G., Bielefeldt-Ohmann H., Wilkinson L., Little M.H., Moritz K.M. (2014). Mid- to late term hypoxia in the mouse alters placental morphology, glucocorticoid regulatory pathways and nutrient transporters in a sex-specific manner. J. Physiol..

[51] Holland O., Dekker Nitert M., Gallo L.A., Vejzovic M., Fisher J.J., Perkins A.V. (2017). Review: Placental mitochondrial function and structure in gestational disorders. Placenta.

[52] Muralimanoharan S., Guo C., Myatt L., Maloyan A. (2015). Sexual dimorphism in miR-210 expression and mitochondrial dysfunction in the placenta with maternal obesity. Int. J. Obes..

[53] Sun T., Gonzalez T.L., Deng N., DiPentino R., Clark E.L., Lee B., Tang J., Wang Y., Stripp B.R., Yao C. (2020). Sexually Dimorphic Crosstalk at the Maternal-Fetal Interface. J. Clin. Endocrinol. Metab..

[54] Hadlock F.P., Harrist R.B., Martinez-Poyer J. (1991). In utero analysis of fetal growth: A sonographic weight standard. Radiology.

[55] Illsley N.P., Wang Z.Q., Gray A., Sellers M.C., Jacobs M.M. (1990). Simultaneous preparation of paired, syncytial, microvillous and basal membranes from human placenta. Biochim. Biophys. Acta.

[56] Napso T., Lean S.C., Lu M., Mort E.J., Desforges M., Moghimi A., Bartels B., El-Bacha T., Fowden A.L., Camm E.J. (2022). Diet-induced maternal obesity impacts feto-placental growth and induces sex-specific alterations in placental morphology, mitochondrial bioenergetics, dynamics, lipid metabolism and oxidative stress in mice. Acta Physiol..

[57] Colleoni F., Morash A.J., Ashmore T., Monk M., Burton G.J., Murray A.J. (2012). Cryopreservation of placental biopsies for mitochondrial respiratory analysis. Placenta.

[58] O’Brien K.A., Gu W., Houck J.A., Holzner L.M.W., Yung H.W., Armstrong J.L., Sowton A.P., Baxter R., Darwin P.M., Toledo-Jaldin L. (2024). Genomic Selection Signals in Andean Highlanders Reveal Adaptive Placental Metabolic Phenotypes That Are Disrupted in Preeclampsia. Hypertension.

[59] Hu Y., Huang K., Sun Y., Wang J., Xu Y., Yan S., Zhu P., Tao F. (2017). Placenta response of inflammation and oxidative stress in low-risk term childbirth: The implication of delivery mode. BMC Pregnancy Childbirth.

[60] Meyuhas O. (2015). Ribosomal Protein S6 Phosphorylation: Four Decades of Research. Int. Rev. Cell Mol. Biol..

[61] Mader S., Lee H., Pause A., Sonenberg N. (1995). The translation initiation factor eIF-4E binds to a common motif shared by the translation factor eIF-4 gamma and the translational repressors 4E-binding proteins. Mol. Cell Biol..

[62] Thoreen C.C., Chantranupong L., Keys H.R., Wang T., Gray N.S., Sabatini D.M. (2012). A unifying model for mTORC1-mediated regulation of mRNA translation. Nature.

[63] Conduit S.E., Zhang C.X.W., Pearce W., Guillermet-Guibert J., Sferruzzi-Perri A.N., Vanhaesebroeck B. (2025). Novel role for PI3Kbeta in placental function through regulation of system A amino acid transporter expression, associated with embryonic lethality. Cell. Mol. Life Sci..

[64] Valenstein M.L., Lalgudi P.V., Gu X., Kedir J.F., Taylor M.S., Chivukula R.R., Sabatini D.M. (2024). Rag-Ragulator is the central organizer of the physical architecture of the mTORC1 nutrient-sensing pathway. Proc. Natl. Acad. Sci. USA.

[65] Egri S.B., Ouch C., Chou H.T., Yu Z., Song K., Xu C., Shen K. (2022). Cryo-EM structures of the human GATOR1-Rag-Ragulator complex reveal a spatial-constraint regulated GAP mechanism. Mol. Cell.

[66] Chun Y., Kim J. (2021). AMPK-mTOR Signaling and Cellular Adaptations in Hypoxia. Int. J. Mol. Sci..

[67] Condon K.J., Orozco J.M., Adelmann C.H., Spinelli J.B., van der Helm P.W., Roberts J.M., Kunchok T., Sabatini D.M. (2021). Genome-wide CRISPR screens reveal multitiered mechanisms through which mTORC1 senses mitochondrial dysfunction. Proc. Natl. Acad. Sci. USA.

[68] Lama-Sherpa T.D., Jeong M.H., Jewell J.L. (2023). Regulation of mTORC1 by the Rag GTPases. Biochem. Soc. Trans..

[69] Hung T.H., Wu C.P., Chen S.F. (2021). Differential Changes in Akt and AMPK Phosphorylation Regulating mTOR Activity in the Placentas of Pregnancies Complicated by Fetal Growth Restriction and Gestational Diabetes Mellitus with Large-For-Gestational Age Infants. Front. Med..

[70] Song L., Wang N., Peng Y., Sun B., Cui W. (2022). Placental lipid transport and content in response to maternal overweight and gestational diabetes mellitus in human term placenta. Nutr. Metab. Cardiovasc. Dis..

[71] McColl E.R., Piquette-Miller M. (2021). Viral model of maternal immune activation alters placental AMPK and mTORC1 signaling in rats. Placenta.

[72] de Mello N.P., Andreotti D.Z., Orellana A.M., Scavone C., Kawamoto E.M. (2020). Inverse sex-based expression profiles of PTEN and Klotho in mice. Sci. Rep..

[73] Wang Y., Buyse J., Courousse N., Tesseraud S., Metayer-Coustard S., Berri C., Schallier S., Everaert N., Collin A. (2020). Effects of sex and fasting/refeeding on hepatic AMPK signaling in chickens (*Gallus gallus*). Comp. Biochem. Physiol. A Mol. Integr. Physiol..

[74] Carey E.A., Albers R.E., Doliboa S.R., Hughes M., Wyatt C.N., Natale D.R., Brown T.L. (2014). AMPK knockdown in placental trophoblast cells results in altered morphology and function. Stem Cells Dev..

[75] Lim R., Barker G., Lappas M. (2015). Activation of AMPK in human fetal membranes alleviates infection-induced expression of pro-inflammatory and pro-labour mediators. Placenta.

[76] Lane S.L., Houck J.A., Doyle A.S., Bales E.S., Lorca R.A., Julian C.G., Moore L.G. (2020). AMP-activated protein kinase activator AICAR attenuates hypoxia-induced murine fetal growth restriction in part by improving uterine artery blood flow. J. Physiol..

[77] Skeffington K.L., Higgins J.S., Mahmoud A.D., Evans A.M., Sferruzzi-Perri A.N., Fowden A.L., Yung H.W., Burton G.J., Giussani D.A., Moore L.G. (2016). Hypoxia, AMPK activation and uterine artery vasoreactivity. J. Physiol..

[78] Griffiths R.M., Pru C.A., Behura S.K., Cronrath A.R., McCallum M.L., Kelp N.C., Winuthayanon W., Spencer T.E., Pru J.K. (2020). AMPK is required for uterine receptivity and normal responses to steroid hormones. Reproduction.

[79] Vyas V., Guerra D.D., Bok R., Powell T., Jansson T., Hurt K.J. (2019). Adiponectin links maternal metabolism to uterine contractility. FASEB J..

[80] Kumagai A., Itakura A., Koya D., Kanasaki K. (2018). AMP-Activated Protein (AMPK) in Pathophysiology of Pregnancy Complications. Int. J. Mol. Sci..

[81] Lorca R.A., Matarazzo C.J., Bales E.S., Houck J.A., Orlicky D.J., Euser A.G., Julian C.G., Moore L.G. (2020). AMPK activation in pregnant human myometrial arteries from high-altitude and intrauterine growth-restricted pregnancies. Am. J. Physiol. Heart Circ. Physiol..

[82] Mahendran D., Donnai P., Glazier J.D., D’Souza S.W., Boyd R.D., Sibley C.P. (1993). Amino acid (system A) transporter activity in microvillous membrane vesicles from the placentas of appropriate and small for gestational age babies. Pediatr. Res..

[83] Shibata E., Hubel C.A., Powers R.W., von Versen-Hoeynck F., Gammill H., Rajakumar A., Roberts J.M. (2008). Placental system A amino acid transport is reduced in pregnancies with small for gestational age (SGA) infants but not in preeclampsia with SGA infants. Placenta.

[84] Glazier J.D., Cetin I., Perugino G., Ronzoni S., Grey A.M., Mahendran D., Marconi A.M., Pardi G., Sibley C.P. (1997). Association between the activity of the system A amino acid transporter in the microvillous plasma membrane of the human placenta and severity of fetal compromise in intrauterine growth restriction. Pediatr. Res..

[85] Paolini C.L., Marconi A.M., Ronzoni S., Di Noio M., Fennessey P.V., Pardi G., Battaglia F.C. (2001). Placental transport of leucine, phenylalanine, glycine, and proline in intrauterine growth-restricted pregnancies. J. Clin. Endocrinol. Metab..

[86] Vaughan O.R., Maksym K., Silva E., Barentsen K., Anthony R.V., Brown T.L., Hillman S.L., Spencer R., David A.L., Rosario F.J. (2021). Placenta-specific Slc38a2/SNAT2 knockdown causes fetal growth restriction in mice. Clin. Sci..

[87] Rosario F.J., Urschitz J., Powell T.L., Brown T.L., Jansson T. (2023). Overexpression of the LAT1 in primary human trophoblast cells increases the uptake of essential amino acids and activates mTOR signaling. Clin. Sci..

[88] Alkhalefah A., Dunn W.B., Allwood J.W., Parry K.L., Houghton F.D., Ashton N., Glazier J.D. (2021). Maternal intermittent fasting during pregnancy induces fetal growth restriction and down-regulated placental system A amino acid transport in the rat. Clin. Sci..

[89] Song L., Sun B., Boersma G.J., Cordner Z.A., Yan J., Moran T.H., Tamashiro K.L.K. (2017). Prenatal high-fat diet alters placental morphology, nutrient transporter expression, and mtorc1 signaling in rat. Obesity.

[90] Davis S.M., Kaar J.L., Ringham B.M., Hockett C.W., Glueck D.H., Dabelea D. (2019). Sex differences in infant body composition emerge in the first 5 months of life. J. Pediatr. Endocrinol. Metab..

[91] Morio H., Reien Y., Hirayama Y., Hashimoto H., Anzai N. (2021). Protein kinase C activation upregulates human L-type amino acid transporter 2 function. J. Physiol. Sci..

[92] Guitart-Mampel M., Juarez-Flores D.L., Youssef L., Moren C., Garcia-Otero L., Roca-Agujetas V., Catalan-Garcia M., Gonzalez-Casacuberta I., Tobias E., Milisenda J.C. (2019). Mitochondrial implications in human pregnancies with intrauterine growth restriction and associated cardiac remodelling. J. Cell. Mol. Med..

[93] Cunningham J.T., Rodgers J.T., Arlow D.H., Vazquez F., Mootha V.K., Puigserver P. (2007). mTOR controls mitochondrial oxidative function through a YY1-PGC-1alpha transcriptional complex. Nature.

[94] Koyanagi M., Asahara S., Matsuda T., Hashimoto N., Shigeyama Y., Shibutani Y., Kanno A., Fuchita M., Mikami T., Hosooka T. (2011). Ablation of TSC2 enhances insulin secretion by increasing the number of mitochondria through activation of mTORC1. PLoS ONE.

[95] Morita M., Gravel S.P., Chenard V., Sikstrom K., Zheng L., Alain T., Gandin V., Avizonis D., Arguello M., Zakaria C. (2013). mTORC1 controls mitochondrial activity and biogenesis through 4E-BP-dependent translational regulation. Cell Metab..

[96] Vaka R., Deer E., Cunningham M., McMaster K.M., Wallace K., Cornelius D.C., Amaral L.M., LaMarca B. (2021). Characterization of Mitochondrial Bioenergetics in Preeclampsia. J. Clin. Med..

[97] Rosario F.J., Barentsen K., Powell T.L., Urschitz J., Brown T.L., Kanai Y., Jansson T. (2024). Trophoblast-specific overexpression of the LAT1 increases transplacental transport of essential amino acids and fetal growth in mice. PNAS Nexus.

