# Supplementary material for: Sexual Dimorphism in Placental mTORC1 Signaling, Amino Acid Transport, and Mitochondrial Respiration at Term
Source: Antioxidants (Basel). 2026 Aug 6;15(8):977. doi: 10.3390/antiox15080977 (PMC13509233; doi:10.3390/antiox15080977)
Supplement: Supplementary file 1 [file antioxidants-15-00977-s001.zip › antioxidants-4377772-supplementary.pdf]

Supplemental Table S1. Primary and secondary antibodies used for Western blot analyses

Target	Species	Dilution of primary antibody	Dilution of secondary antibody	Manufacturer	Catalogue number
S6RP (Ser-235/236)	rabbit	1:1000	1:3000	Cell Signaling	#2211
S6RP	rabbit	1:1000	1:3000	Cell Signaling	#2217
4E-BP1(Thr-37/46)	rabbit	1:1000	1:3000	Cell Signaling	#9459
4E-BP1	rabbit	1:1000	1:3000	Cell Signaling	#9452
OGT	rabbit	1:1000	1:3000	Cell Signaling	#23177
AKT (Ser-473)	rabbit	1:500	1:3000	Cell Signaling	#9271
AKT (Thr-308)	rabbit	1:500	1:3000	Cell Signaling	#4056
AKT	rabbit	1:500	1:3000	Cell Signaling	#9272
AMPK (Thr-172)	rabbit	1:1000	1:3000	Cell Signaling	#2535
AMPK	rabbit	1:1000	1:3000	Cell Signaling	#2532
ERK1/2 p44/42 (Thr-202/Tyr-204)	rabbit	1:1000	1:3000	Cell Signaling	#4370
ERK1/2 p44/42	rabbit	1:1000	1:3000	Cell Signaling	#4695
OXPHOS	mouse	1:1000	1:3000	Abcam	ab110413
SNAT1	rabbit	1:3000	1:1000	Proteintech	286321
SNAT2	rabbit	1:5000	1:1000	Kind gift from Dr. Puttur Prasad	
SNAT4	rabbit	1:1000	1:3000	Eurogentec	
β-actin	rabbit	1:1000	1:3000	Cell Signaling	#4970

Abbreviations: S6RP, S6 ribosomal protein; Ser, Serine; 4EBP1, 4E-binding protein 1; OGT, O-GlcNAc transferase; Thr, threonine; AKT, protein kinase B; AMPK, 5' AMP-activated protein kinase; ERK, Extracellular signal-regulated kinase; OXPHOS, oxidative phosphorylation; SNAT, Sodium-coupled neutral amino acid transporter.

Supplemental Table S2. Sequential antibody probing following membrane stripping in Western blot analyses

	Initial antibody	Stripped and reprobed with
Figure 1A	4E-BP1(Thr-37/46)	S6RP (Ser-235/236)
Figure 4A	AKT (Thr-308)	OGT

Supplemental Table S3. Selected clinical characteristics of subjects included in MVM amino acid transport experiments

	Female fetuses (n=10)	Male fetuses (n=10)	*p* value
Maternal age (years)	31.60±1.70<br>[23-40]	30.60±1.47<br>[23-36]	0.66
BMI (kg/m^2)	22.51±0.61<br>[18.90-25.00]	23.22±0.63<br>[20.20-25.70]	0.43
Gestational age (weeks)	39.24±0.22<br>[37.86-40.29]	39.50±0.25<br>[38.86, 41.29]	0.44
Birth weight (g)	3178.70±99.79<br>[2718.00-3800.00]	3425.70±99.94<br>[3015.00-3860.00]	0.10
Birth weight z score	-0.23±0.20<br>[-1.20-1.03]	-0.09±0.16<br>[-0.74-0.70]	0.60
Placental weight (g)	606.79±35.45<br>[437.00-873.43]	566.40±33.25<br>[388.60-697.00]	0.41
Birthweight-to-placental weight ratio	5.33±0.21<br>[3.77-6.22]	6.20±0.31<br>[3.77-6.22]	**0.04**
Delivery mode (C/V)	6/4	7/3	1.00
**Race**			
White	9 (90%)	7 (70%)	1.00
Black	1 (10%)	1 (10%)	
Asian	-	2 (20%)	
**Ethnicity**			
Hispanic	1 (10%)	1 (10%)	1.00
Non-Hispanic	9 (90%)	9 (90%)	

Data are presented as means ± SEM and range [minimum- maximum]. Abbreviations: MVM, microvillous membrane; n, numbers; C, caesarean section; V, vaginal. Statistical significance of differences between female and male groups were assessed using unpaired Student's t-test except for Delivery mode. Statistical significance of the difference of delivery mode between female and male groups and ethnicity was assessed using Fisher's test.

Supplemental Table S4. Selected clinical characteristics for subjects included in placental oxygen consumption rate experiments

	Female fetuses (n=12)	Male fetuses (n=8)	*p* value
Maternal age (years)	30.42±1.74<br>[23-41]	28.75±2.59<br>[18-37]	0.59
BMI (kg/m^2)	24.83±0.98<br>[18.97-31.90]	22.86±1.00<br>[18.90-27.00]	0.19
Gestational age (weeks)	39.37±0.26<br>[37.29-40.86]	39.23±0.10<br>[39.00-39.71]	0.61
Birth weight (g)	3325.00±120.10<br>[4055-2875]	3315.63±79.66<br>[3035-3685]	0.95
Birth weight z score	0.02±0.19<br>[-0.89-1.12]	-0.21±0.17<br>[-0.85-0.47]	0.42
Placental weight (g)	651.40 ±31.99<br>[821.65-509.60]	625.71±35.13<br>[512.13-804.00]	0.60
Birthweight-to-placental weight ratio	5.08±0.23<br>[4.28-6.26]	5.38±0.25<br>[4.41-6.34]	0.38
Delivery mode (C/V)	7/5	3/2	1.00
**Race**			
White	7 (58%)	5 (63%)	1.00
Asian	-	1 (12%)	
Unknown	5 (42%)	2 (25%)	
**Ethnicity**			
Hispanic	4 (33%)	5 (63%)	0.35
Non-Hispanic	8 (67%)	3 (37%)	

Data are presented as means ± SEM and range [minimum-maximum].

Abbreviations: n, numbers; C, caesarean section; V, vaginal.

Statistical significance of differences between female and male groups were assessed using unpaired Student's t-test except for Delivery mode. Statistical significance of the difference of Delivery mode between female and male groups and ethnicity was assessed using Fisher's test.

Supplemental Table S5. Outliers identified by Grubbs' test in placental signaling Western blot analyses.

Figure	Protein	Group	Relative density	Test
Figure 1A	S6RP (Ser-235/236)	Male	6.2	Grubbs' test
Figure 1D	Total 4E-BP1	Male	3.4 and 2.1	Grubbs' test
Figure 2B	Total AMPK	Male	2.8	Grubbs' test
Figure 3A	Akt-Thr-308	Female	3.9	Grubbs' test
Figure 3B	Akt-Ser-473	Male	3.1	Grubbs' test
Figure 5 D	SNAT4	Male	4.5	Grubbs' test
		Female	2.0	Grubbs' test
Figure 8 D	Mitochondrial electron transport chain complex I	Female	4.4	Grubbs' test
Figure 8 F	Mitochondrial electron transport chain complex III	Female	3.0 and 2.5	Grubbs' test
Figure 8 G	Mitochondrial electron transport chain complex IV	Male	2.8	Grubbs' test
		Female	3.1 and 2.5	Grubbs' test

Supplemental Table S6. Assessment of the effect of delivery mode (vaginal versus cesarean delivery) on placental signaling pathway readouts.

	**Female** (Vaginal vs cesarean delivery)			**Male** (Vaginal vs cesarean delivery)		
Variable	Normally distributed?	Statistical Test	P-Value	Normally distributed?	Statistical Test	P-Value
S6RP (Ser-235/236)	Yes	Unpaired Student's t-test	0.33	Yes	Unpaired Student's t-test	0.17
S6RP	Yes	Unpaired Student's t-test	0.24	Yes	Unpaired Student's t-test	0.78
4E-BP1(Thr-37/46)	Yes	Unpaired Student's t-test	0.48	Yes	Unpaired Student's t-test	0.12
4E-BP1	No	Mann–Whitney U test	0.33	No	Mann–Whitney U test	0.64
OGT	Yes	Unpaired Student's t-test	0.87	Yes	Unpaired Student's t-test	0.95
AKT (Ser-473)	Yes	Unpaired Student's t-test	0.72	Yes	Unpaired Student's t-test	0.87
AKT (Thr-308)	Yes	Unpaired Student's t-test	0.09	Yes	Unpaired Student's t-test	0.76
AKT	No	Mann–Whitney U test	0.15	Yes	Unpaired Student's t-test	0.62
AMPK (Thr-172)	Yes	Unpaired Student's t-test	0.72	Yes	Unpaired Student's t-test	0.54
AMPK	No	Mann–Whitney U test	**0.03**	No	Mann–Whitney U test	0.98
ERK1/2 p44/42 (Thr-202/Tyr-204)	No	Mann–Whitney U test	0.68	No	Mann–Whitney U test	0.17
ERK1/2 p44/42	Yes	Unpaired Student's t-test	0.84	Yes	Unpaired Student's t-test	0.06
The ratio of AKT(Thr-308) to total AKT	Yes	Unpaired Student's t-test	0.58	Yes	Unpaired Student's t-test	0.48
The ratio of AKT(Ser-473) to total AKT	Yes	Unpaired Student's t-test	0.61	No	Mann–Whitney U test	0.74
The ratio of AMPK (Thr-172) to total AMPK	No	Mann–Whitney U test	0.17	No	Mann–Whitney U test	0.95
The ratio of S6RP (Ser-235/236) to total S6RP	Yes	Unpaired Student's t-test	0.77	No	Mann–Whitney U test	0.19
The ratio of 4E-BP1(Thr-37/46) to total 4EBP1	No	Mann–Whitney U test	0.15	No	Mann–Whitney U test	0.36
The ratio of ERK1/2 p44/42 (Thr-202/Tyr-204) to ERK1/2 p44/42	No	Mann–Whitney U test	0.64	No	Mann–Whitney U test	0.06

Data normality was assessed using the Shapiro–Wilk normality test. For variables that were normally distributed in one or both the vaginal and cesarean delivery groups, differences were analyzed using an unpaired Student's t-test. For variables that were not normally distributed in one or both groups, differences were analyzed using the Mann–Whitney U test. P values represent comparisons between vaginal and cesarean delivery groups.

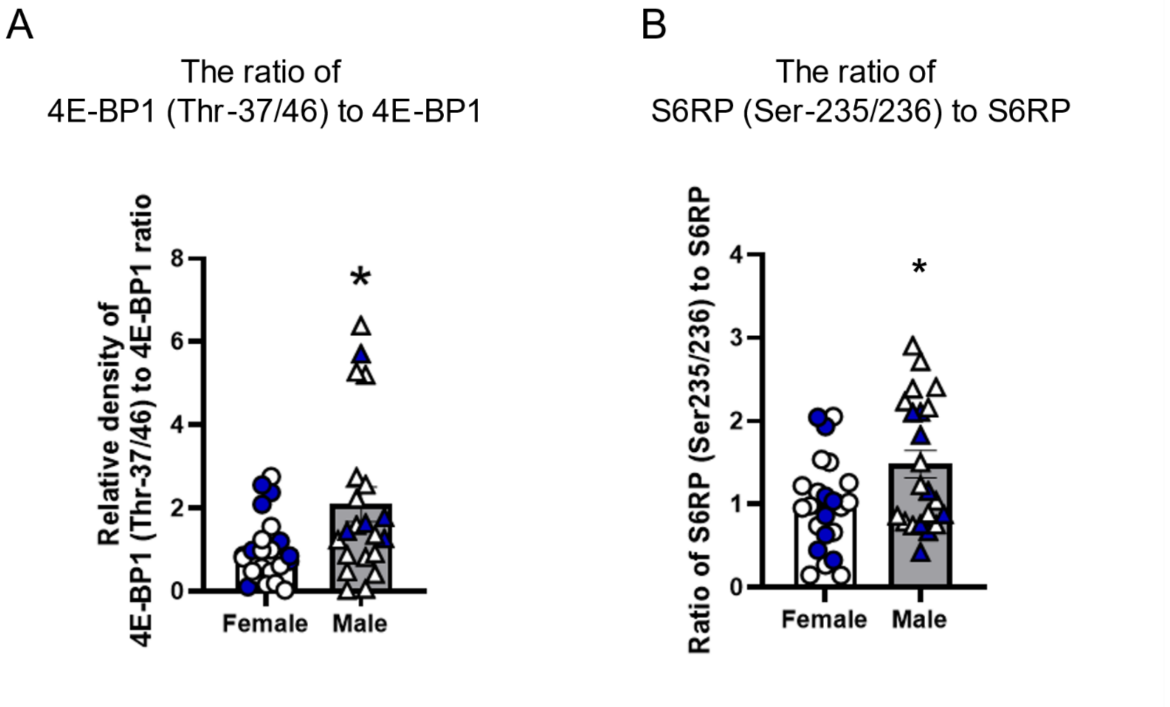

Supplemental Figure S1. Ratios of phosphorylated 4E-BP1 and S6RP to total protein expression in female and male placentas

Quantification of the ratio of phosphorylated 4E-BP1 (Thr-37/46) to total 4E-BP1 protein expression (A) and the ratio of phosphorylated S6RP (Ser-235/236) to total S6RP protein expression (B) in homogenates from female and male term placentas. Data are presented as means ± SEM. *$p < 0.05$ versus female placentas. Statistical analyses were performed using unpaired Student's t-test or Mann–Whitney test. One outlier (male sample) was removed from phosphorylated S6RP (Ser235/236) analyses, and two outliers (male samples) were removed from total 4E-BP1 analyses using Grubbs' test ($\alpha = 0.05$). No outliers were identified for total S6RP or phosphorylated 4E-BP1 analyses. Final sample sizes were n = 21–22 female placentas and n = 22–23 male placentas depending on the analysis. Data shown in panel B was normally distributed and statistical difference was evaluated using an unpaired Student's t-test. Data shown in panel A was not normally distributed and was analyzed using the Mann–Whitney test. Abbreviations: 4E-BP1, eukaryotic initiation factor 4E-binding protein 1; S6RP, S6 ribosomal protein; Ser, Serine; Thr, Threonine. To illustrate the distribution of delivery modes within each sex, samples from vaginal deliveries are highlighted in blue, whereas black symbols indicate placentas delivered by cesarean section.

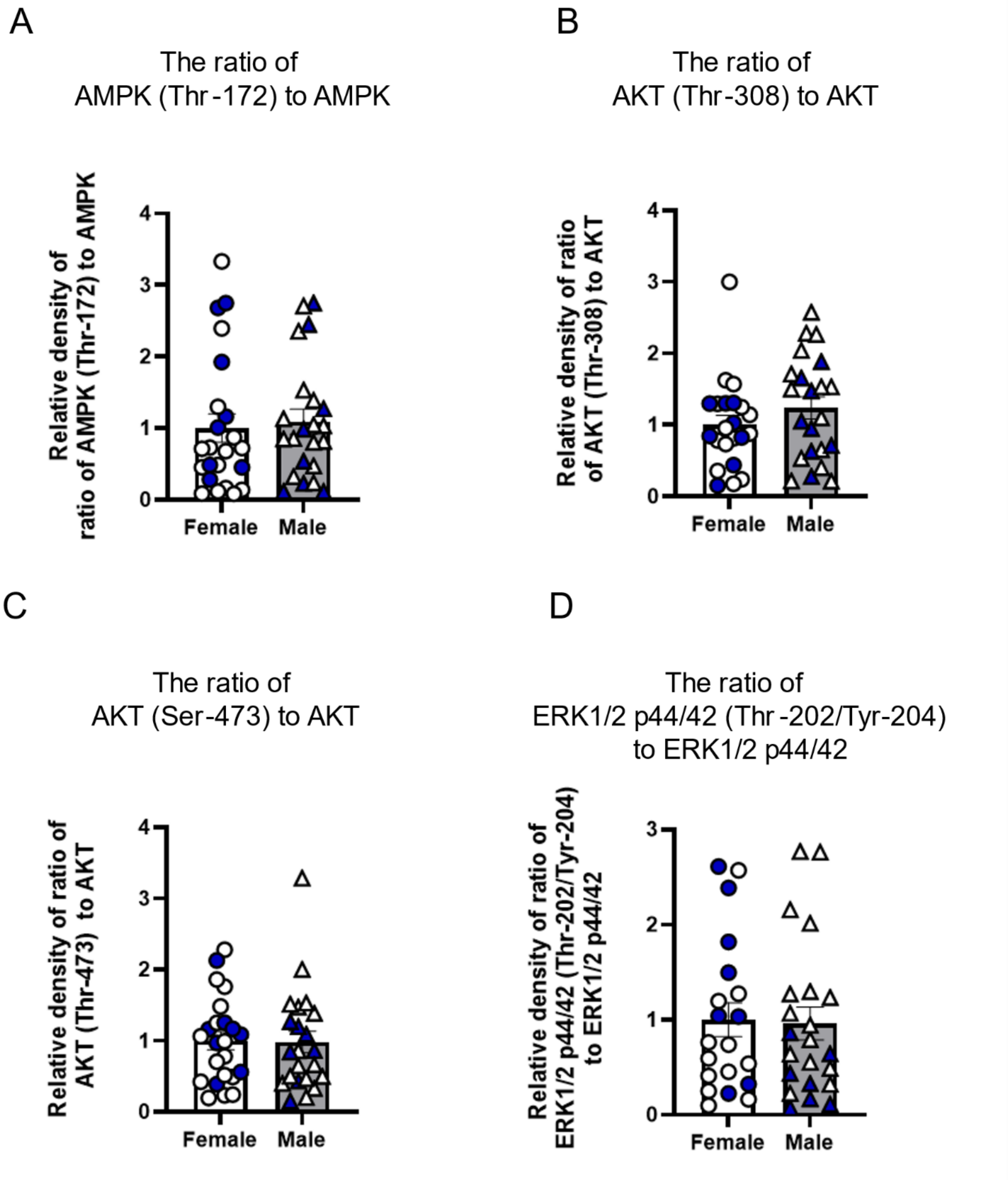

Supplemental Figure S2. Ratios of phosphorylated AMPK, AKT, and ERK1/2 to total AMPK, AKT, and ERK1/2 protein expression in female and male placentas

Quantification of the ratio of phosphorylated AMPK (Thr-172) to total AMPK protein expression (A), phosphorylated AKT (Thr308) to total AKT protein expression (B), phosphorylated AKT (Ser473) to total AKT protein expression (C), and phosphorylated ERK1/2 p44/42 (Thr-202/Tyr-204) to total ERK1/2 p44/42 protein expression (D) in homogenates from female and male term

placentas. Data is presented as means ± SEM. Statistical analyses were performed using unpaired Student's t-test or Mann–Whitney test. One outlier (male sample) was removed from the AMPK phosphorylation/total AMPK ratio analysis using Grubbs' test (α = 0.05). No outliers were identified for the AKT (Thr-308), AKT (Ser-473), or ERK1/2 ratio analyses. Final sample sizes were n = 23 female placentas and n = 22–23 male placentas depending on the analysis. Data shown in panels B-C were normally distributed and statistical differences were evaluated using an unpaired Student's t-test. Data shown in panels A and D were not normally distributed and were analyzed using the Mann–Whitney test. Abbreviations: AMPK, 5′ AMP-activated protein kinase; AKT, protein kinase B; ERK, extracellular signal-regulated kinase; Ser, serine; Thr, threonine; Tyr, tyrosine. To illustrate the distribution of delivery modes within each sex, samples from vaginal deliveries are highlighted in blue, whereas black symbols indicate placentas delivered by cesarean section.

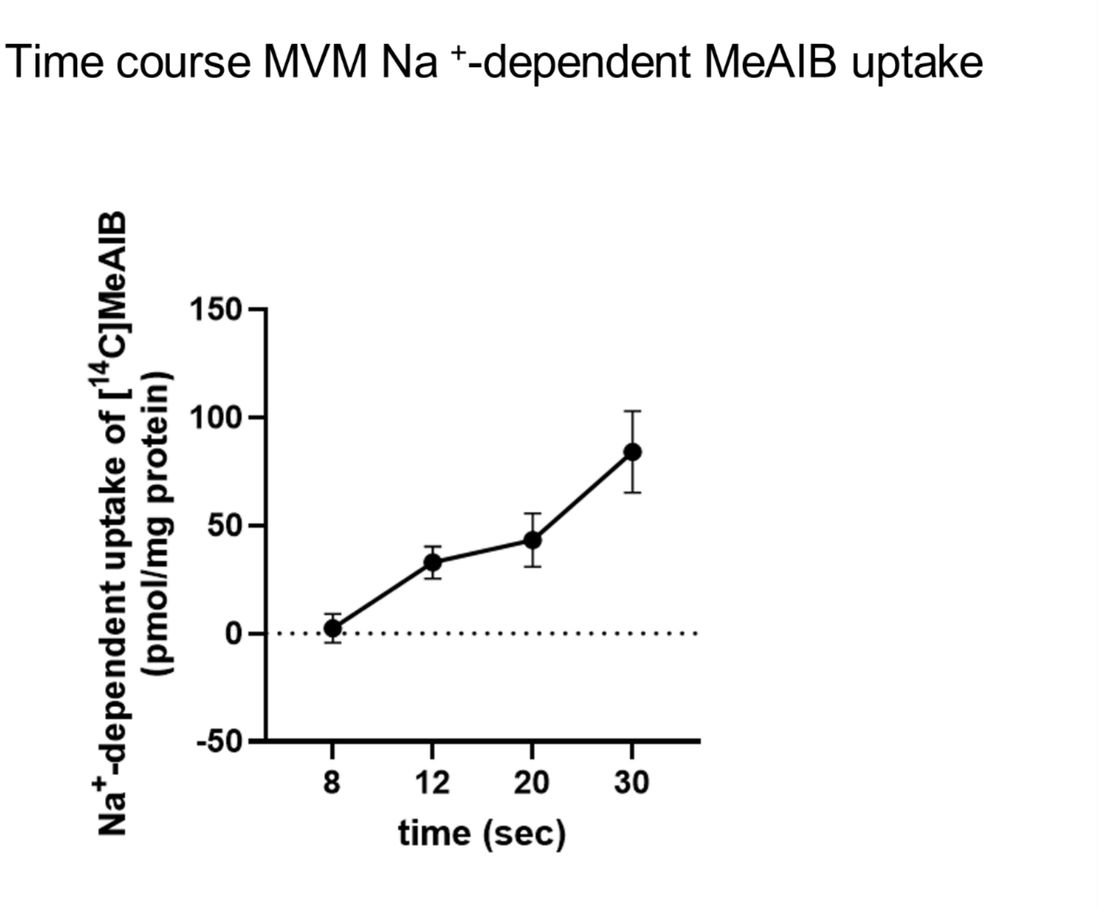

Supplemental Figure S3. Time course of Na$^+$-dependent MeAIB uptake in placental MVM vesicles

Time-course analysis of Na$^+$-dependent methylaminoisobutyric acid (MeAIB) uptake in microvillous membrane (MVM) vesicles isolated from term human placentas. Uptake was measured at 8, 12, 20, and 30 s and expressed as pmol/mg protein. Data are presented as means ± SEM. The 20 s time point was selected for subsequent transport experiments as it represented initial rate uptake conditions. Abbreviations: MVM, microvillous membrane; MeAIB, methylaminoisobutyric acid; Sec, second.

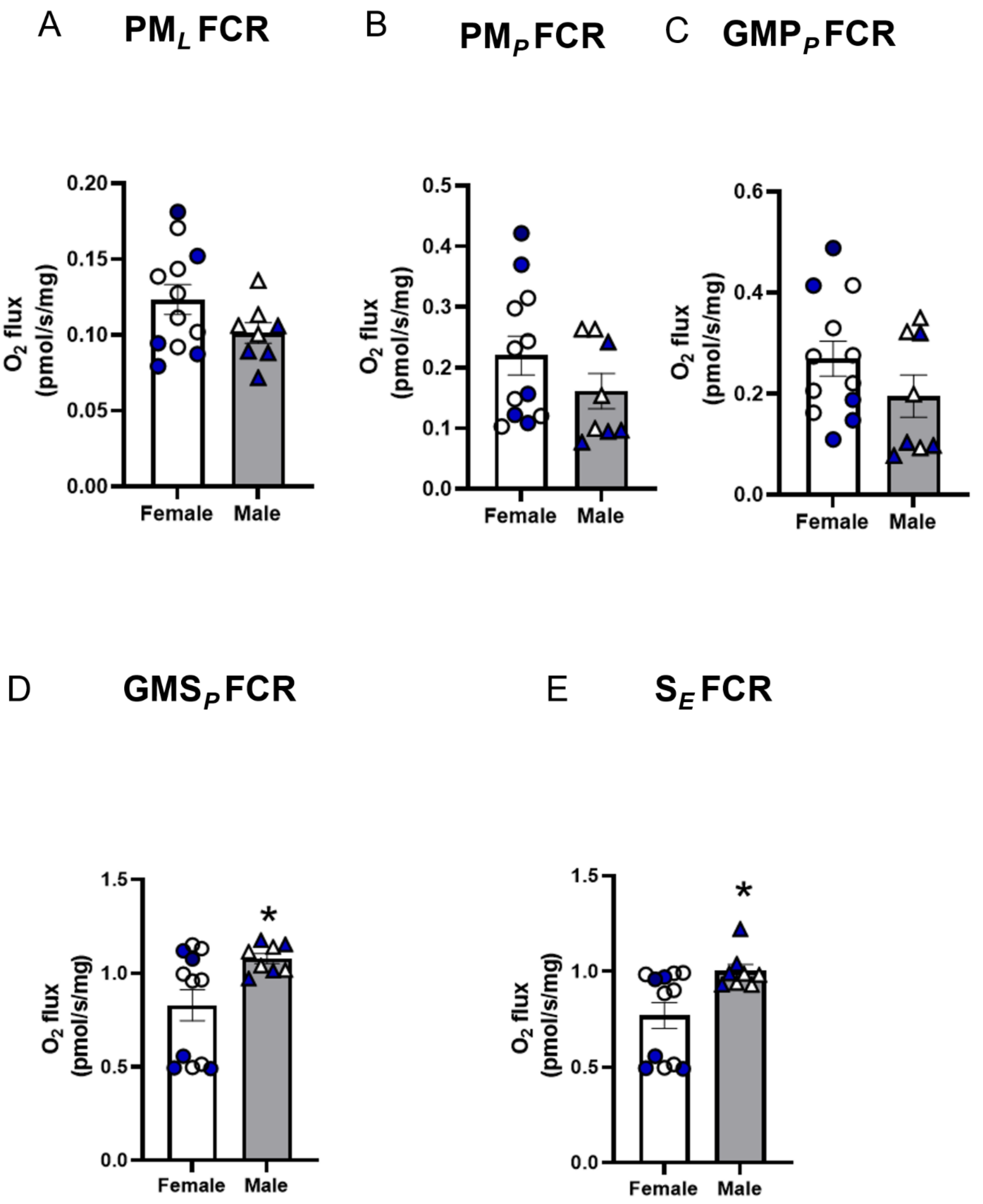

Supplemental Figure S4. Flux control ratios of placental mitochondrial respiratory states assessed using the Carb SUIT protocol in female and male placentas.

Flux control ratios (FCRs) of mitochondrial respiratory states measured in term placental villous

tissue using the carbohydrate substrate–uncoupler–inhibitor titration (Carb SUIT) protocol. FCRs were calculated by normalizing each respiratory state to maximal uncoupled electron transport system capacity (GMS_E). Basal LEAK respiration supported by malate and pyruvate (PM_L FCR) (A); ADP-stimulated oxidative phosphorylation through complex I (PM_P FCR) (B); glutamate-supported complex I oxidative phosphorylation (GMP_P FCR) (C); maximal oxidative phosphorylation supported by convergent electron flow through complexes I and II following succinate addition (GMS_P FCR) (D); and complex II-supported respiration following rotenone inhibition of complex I (S_E FCR) (E). Data are presented as means ± SEM. $p < 0.05$ versus female placentas. Data shown in panels A (PM_L FCR) and B (PM_P FCR) were normally distributed and statistical differences were evaluated using an unpaired Student's *t*-test. Data shown in panels C (GMP_P FCR), D (GMS_P FCR), and E (S_E FCR) were not normally distributed and were analyzed using the Mann–Whitney test. Final sample sizes were n = 12 female placentas and n = 8 male placentas. Abbreviations: FCR, flux control ratio; PM_L, pyruvate/malate LEAK respiration; PM_P, pyruvate/malate oxidative phosphorylation; GMP_P, glutamate-supported oxidative phosphorylation; GMS_P, glutamate/malate/succinate oxidative phosphorylation; S_E, succinate-supported respiration. Flux control ratios were calculated using ROX-corrected respiratory fluxes, with each respiratory state expressed relative to the ROX-corrected maximal electron transport system capacity (GMS_E).

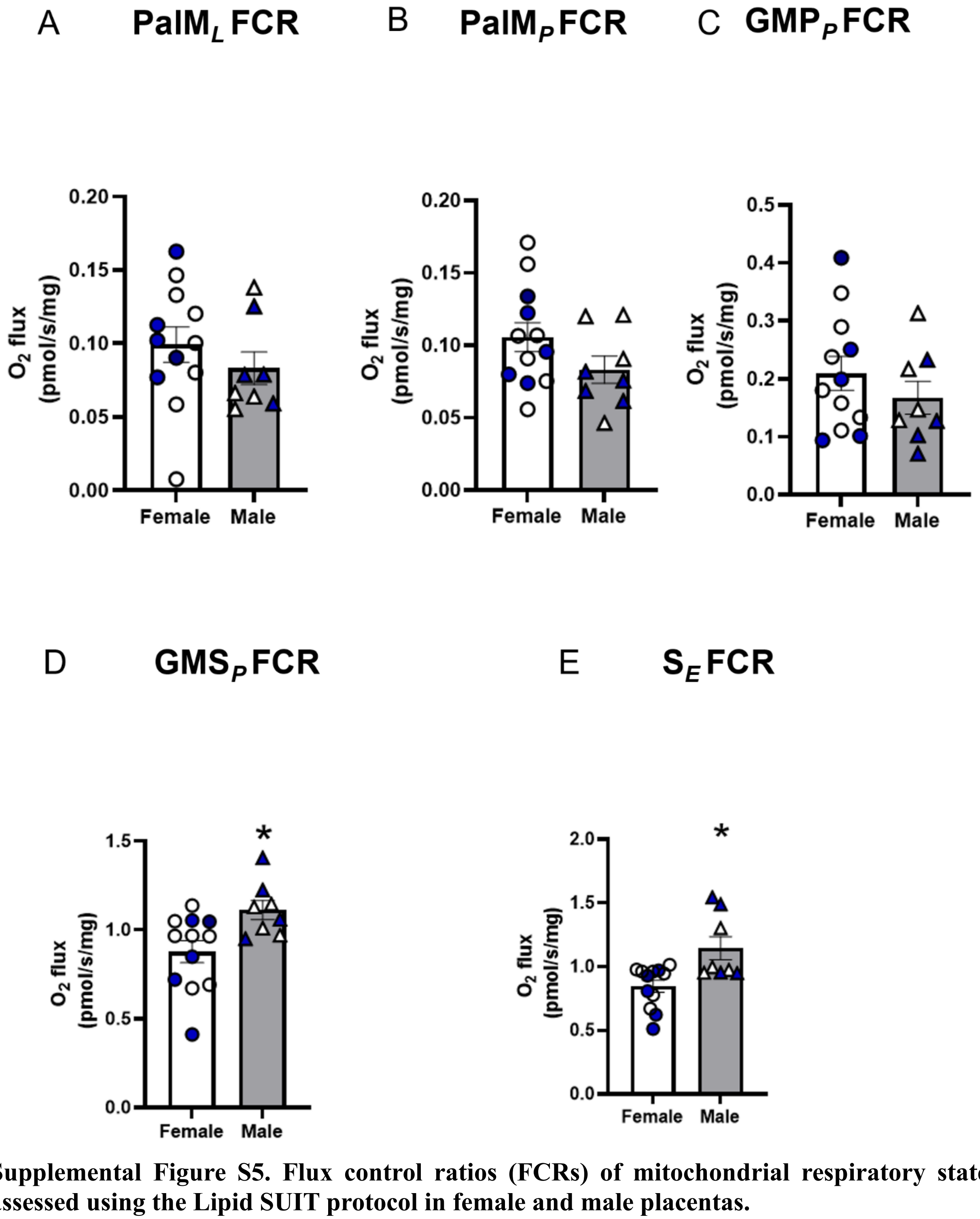

Supplemental Figure S5. Flux control ratios (FCRs) of mitochondrial respiratory states assessed using the Lipid SUIT protocol in female and male placentas.

Flux control ratios (FCRs) of mitochondrial respiratory states in term placental villous tissue

measured using the lipid substrate–uncoupler–inhibitor titration (Lipid SUIT) protocol. FCRs were calculated by normalizing each respiratory state to the maximal uncoupled electron transport system (ETS) capacity (GMS_E). β-oxidation-linked LEAK respiration supported by palmitoyl-carnitine and malate ($PalM_L$) (A); ADP-stimulated β-oxidation-supported oxidative phosphorylation with palmitoyl-carnitine and malate ($PalM_P$) (B); glutamate-supported complex I oxidative phosphorylation (GMP_P) (C); maximal oxidative phosphorylation capacity following succinate addition, reflecting convergent complex I and II electron flow (GMS_P) (D); and complex II-supported respiration following rotenone inhibition of complex I (S_E) (E). Data are presented as means ± SEM. $p < 0.05$ versus female placentas. Final sample sizes were n = 12 female placentas and n = 8 male placentas. Data shown in panels A and E were not normally distributed and statistical differences were evaluated using the Mann–Whitney test. Data shown in panels B–D were normally distributed and statistical differences were evaluated using an unpaired Student's *t*-test. Abbreviations: FCR, flux control ratio; PalML, palmitoyl-carnitine/malate LEAK respiration; PalMP, palmitoyl-carnitine/malate oxidative phosphorylation; GMPP, glutamate-supported oxidative phosphorylation; GMSP, glutamate/malate/succinate oxidative phosphorylation; SE, succinate-supported respiration; ETS, electron transport system. Flux control ratios were calculated using ROX-corrected respiratory fluxes, with each respiratory state expressed relative to the ROX-corrected maximal electron transport system capacity (GMS_E).

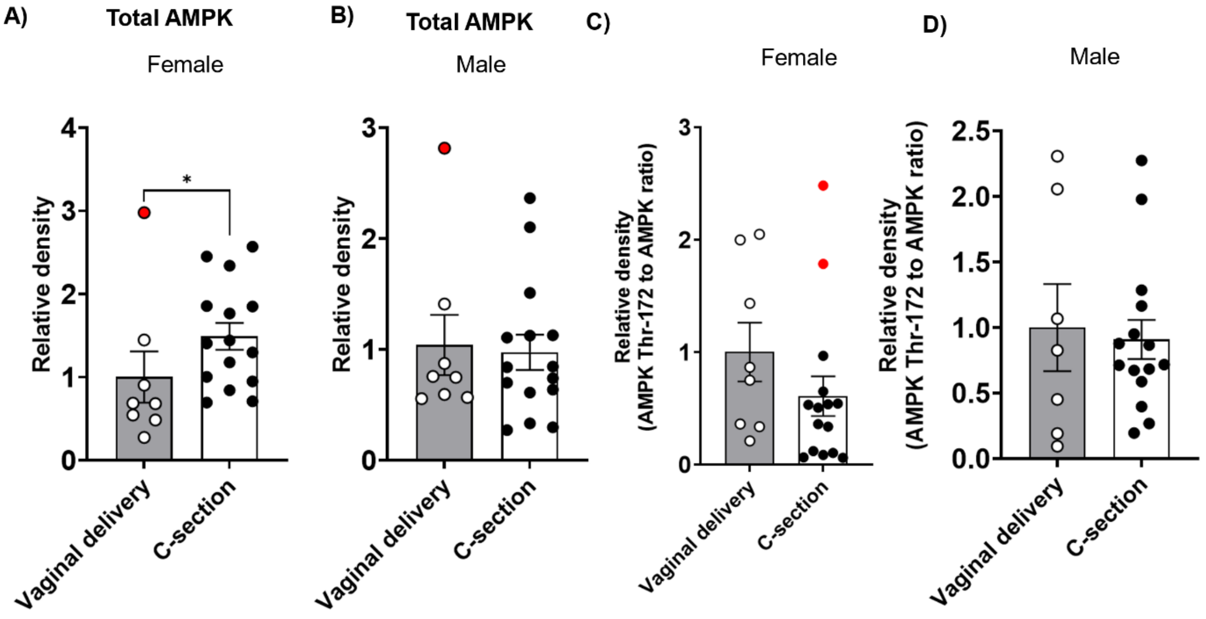

Supplemental Figure S6. Effect of mode of delivery on AMPK protein expression and phosphorylated AMPK/total AMPK ratio in female and male placentas.

Comparison of total AMPK protein expression in placentas delivered by vaginal delivery or cesarean section, analyzed separately for female (A) and male (B) placentas. The ratio of phosphorylated AMPK (Thr172) to total AMPK protein expression was also compared between vaginal and cesarean deliveries in female (C) and male (D) placentas. Histogram bars summarize densitometric analyses of Western blots. For each analysis, the mean density of placentas delivered by vaginal delivery within each sex was assigned an arbitrary value of 1.0. Data are presented as means ± SEM. *$p < 0.05$ versus vaginal delivery. Statistical analyses were performed using the Mann–Whitney U test. One outlier was identified in each of the female and male vaginal delivery

groups for the total AMPK analysis using Grubbs' test ($\alpha = 0.05$). Both outliers were retained and included in the statistical analysis. No outliers were identified for the phosphorylated AMPK (Thr172)/total AMPK ratio analyses. Final sample sizes for total AMPK protein expression were female: vaginal delivery (n = 8), cesarean section (n = 15); male: vaginal delivery (n = 8), cesarean section (n = 15). Final sample sizes for the phosphorylated AMPK (Thr-172)/total AMPK ratio were female: vaginal delivery (n = 8), cesarean section (n = 15); male: vaginal delivery (n = 7), cesarean section (n = 15). Abbreviations: AMPK, 5′ AMP-activated protein kinase; Thr, threonine.

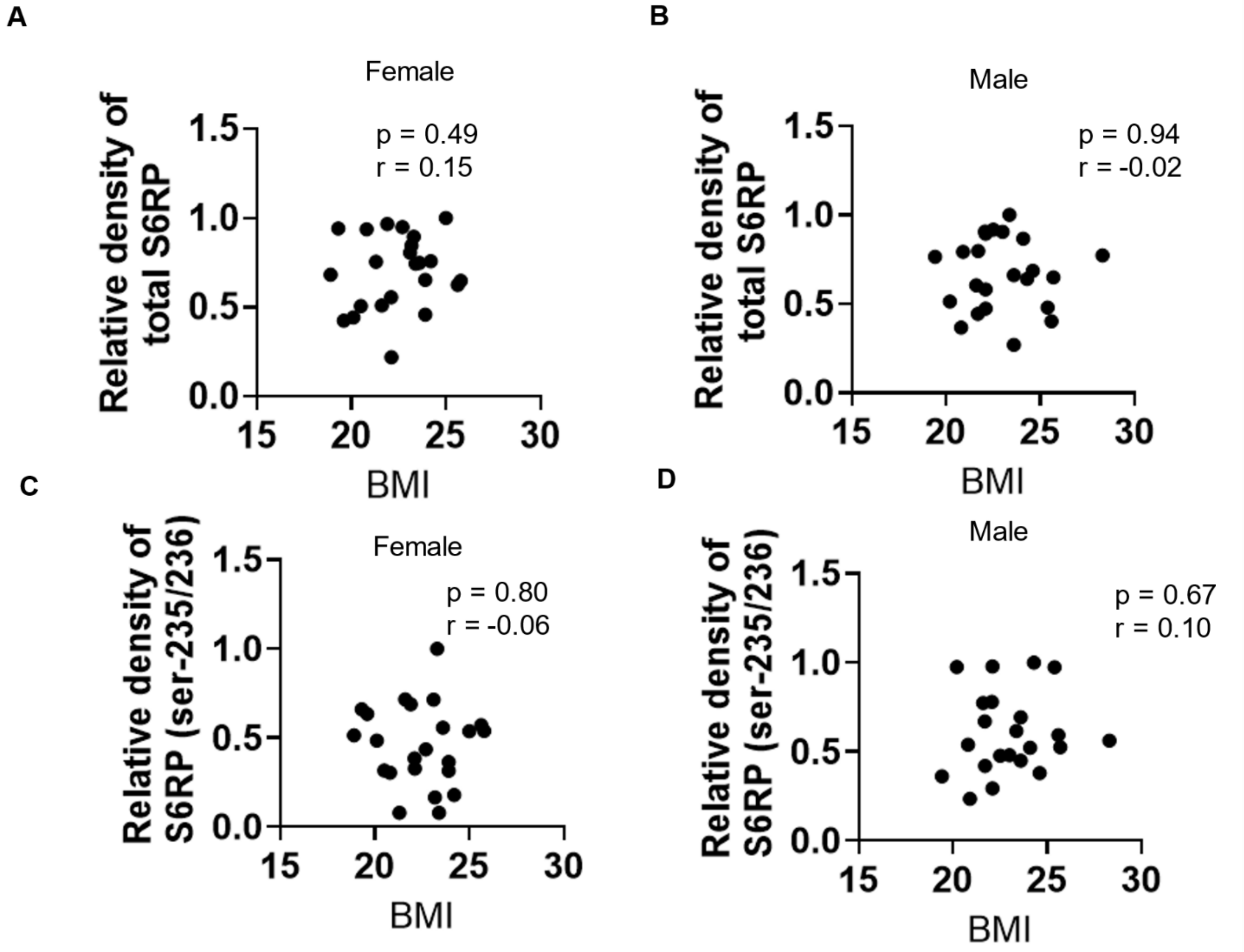

Supplemental Figure S7. Correlation between maternal BMI and S6RP protein expression/phosphorylation in female and male placentas

Correlation analyses between maternal BMI and total S6RP protein expression in female (A) and male (B) placentas, and phosphorylated S6RP (Ser-235/236) protein expression in female (C) and male (D) placentas. Each symbol represents an individual placenta. Pearson's correlation coefficient (r) and corresponding p value are shown for each analysis. No significant correlations were observed. Values are presented as means ± SEM. Statistical significance was defined as $p < 0.05$. Abbreviations: S6RP, S6 ribosomal protein; Ser, serine; BMI, body mass index; r, Pearson correlation coefficient.

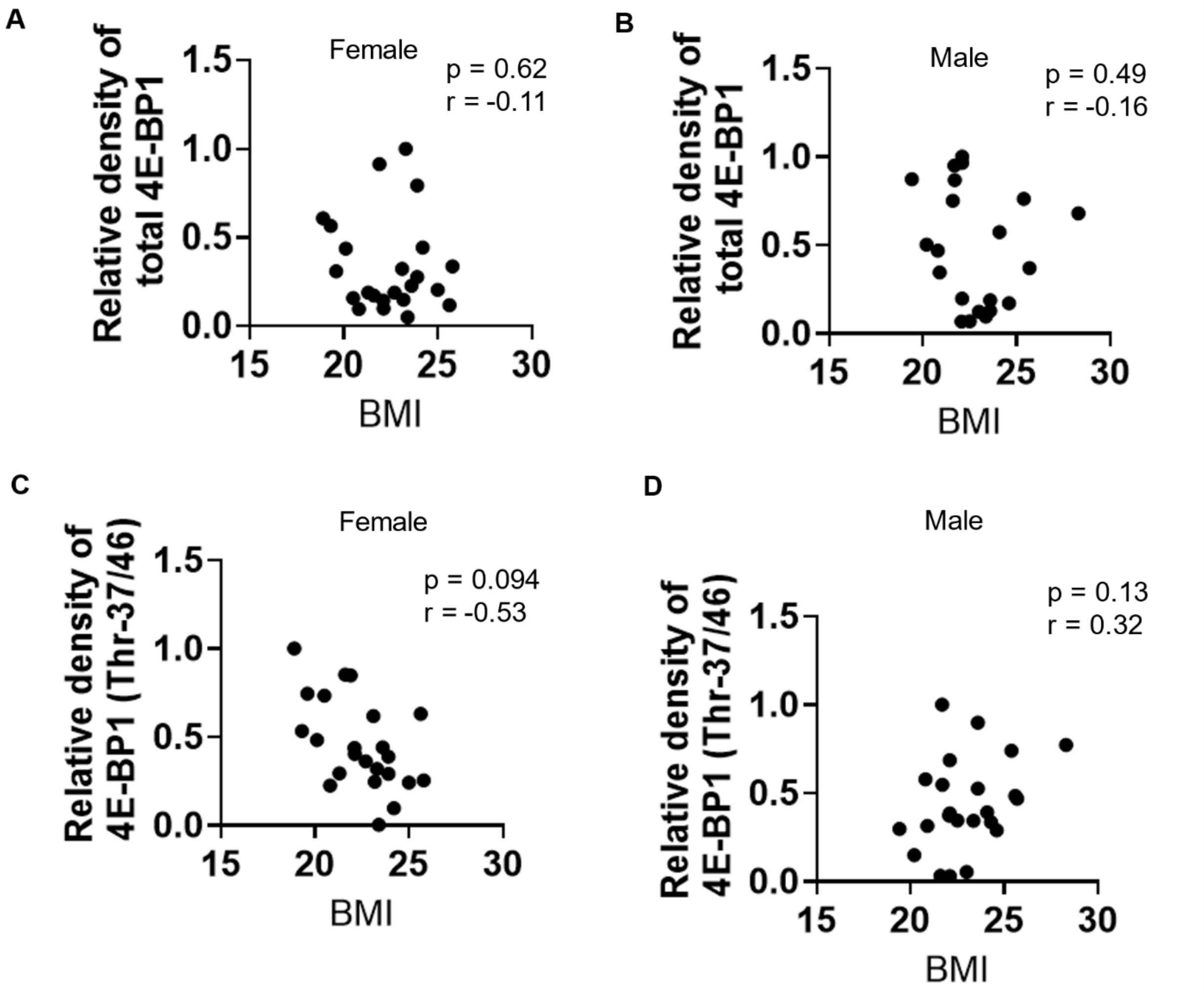

Supplemental Figure S8. Correlation between maternal BMI and 4E-BP1 protein expression/phosphorylation in female and male placentas

Correlation analyses between maternal BMI and total 4E-BP1 protein expression in female (A) and male (B) placentas, and phosphorylated 4E-BP1 (Thr-37/46) protein expression in female (C) and male (D) placentas. Each symbol represents an individual placenta. Pearson's correlation coefficient (r) and corresponding p value are shown for each analysis. No significant correlations were observed. Values are presented as means ± SEM. Statistical significance was defined as $p < 0.05$. Abbreviations: 4E-BP1, eukaryotic initiation factor 4E-binding protein 1; Thr, threonine; BMI, body mass index; r, Pearson correlation coefficient.

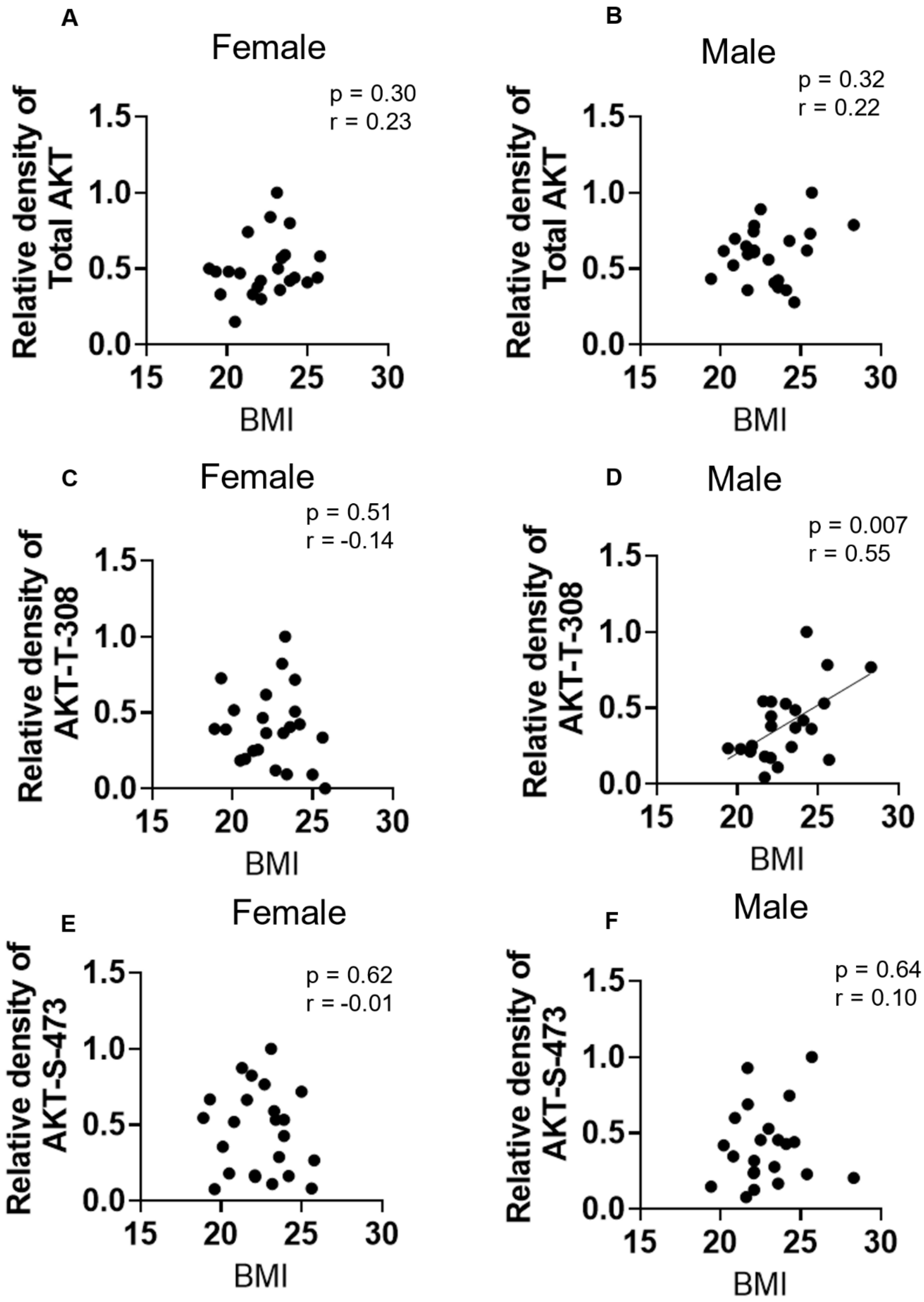

Supplemental Figure S9. Correlation between maternal BMI and AKT protein expression /phosphorylation in female and male placentas

Correlation analyses between maternal BMI and total AMPK protein expression in female (A) and

male (B) placentas, and phosphorylated AMPK (Thr172) protein expression in female (C) and male (D) placentas. Each symbol represents an individual placenta. Pearson's correlation coefficient (**r**) and corresponding **p** value are shown for each analysis. No significant correlations were observed. Values are presented as means ± SEM. Statistical significance was defined as **p < 0.05**. Abbreviations: AMPK, 5′ AMP-activated protein kinase; Thr, threonine; BMI, body mass index; **r**, Pearson correlation coefficient.

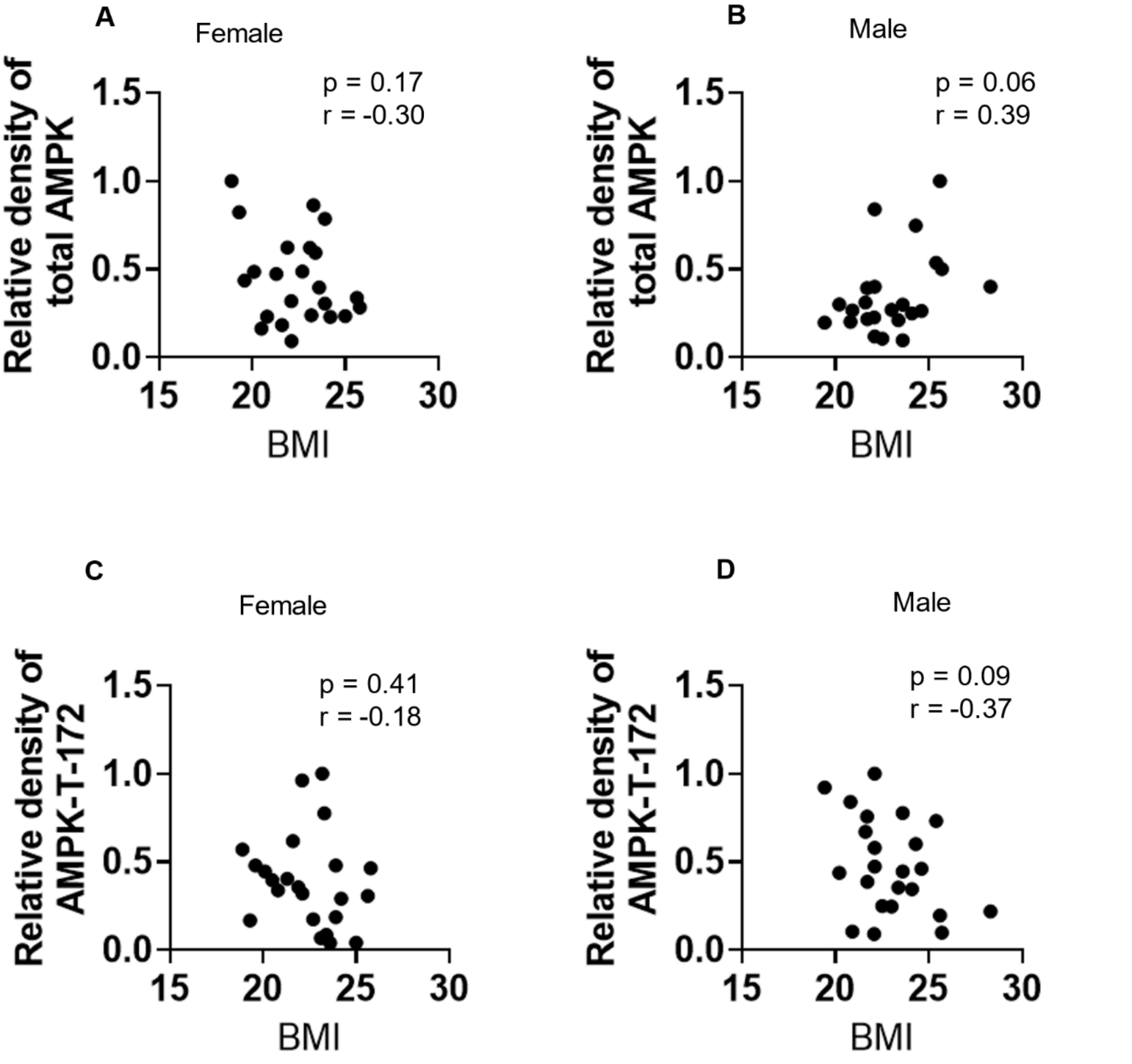

Supplemental Figure S10. Correlation between maternal BMI and AMPK protein expression/phosphorylation in female and male placentas

Correlation analyses between maternal BMI and total AKT protein expression in female (A) and male (B) placentas, phosphorylated AKT (Thr-308) protein expression in female (C) and male (D) placentas, and phosphorylated AKT (Ser-473) protein expression in female (E) and male (F) placentas. Each symbol represents an individual placenta. Pearson's correlation coefficient (r) and corresponding p value are shown for each analysis. Values are presented as means ± SEM. Statistical significance was defined as $p < 0.05$. Abbreviations: AKT, protein kinase B; Thr, threonine; Ser, serine; BMI, body mass index; r, Pearson correlation coefficient.

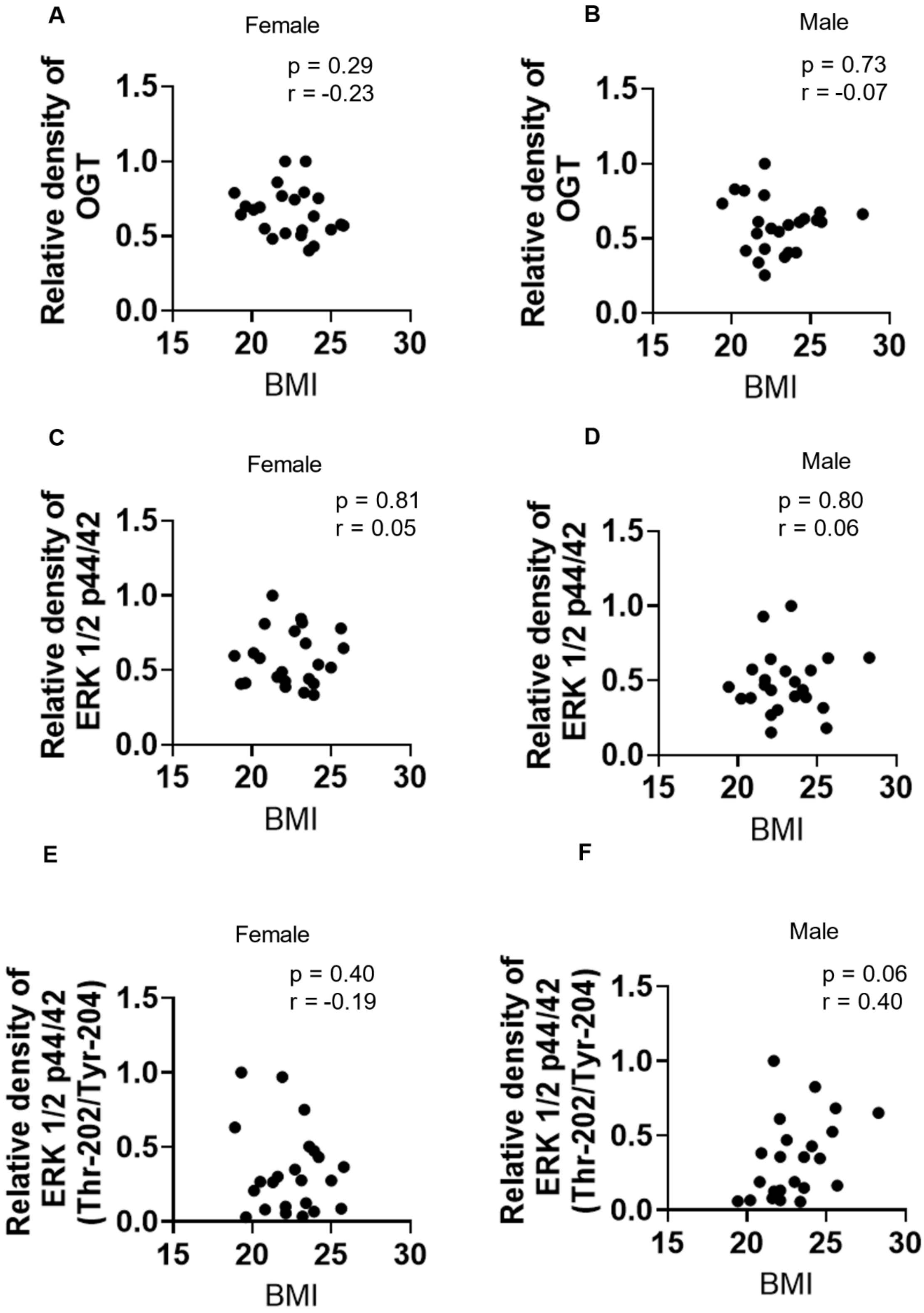

Supplemental Figure S11. Correlation between maternal BMI and OGT and ERK1/2 protein expression-phsophorylation in female and male placentas

Correlation analyses between maternal BMI and OGT protein expression in female (A) and male

(B) placentas, total ERK1/2 p44/42 protein expression in female (C) and male (D) placentas, and phosphorylated ERK1/2 p44/42 (Thr202/Tyr204) protein expression in female (E) and male (F) placentas. Each symbol represents an individual placenta. Pearson's correlation coefficient (r) and corresponding p value are shown for each analysis. No significant correlations were observed. Values are presented as means ± SEM. Statistical significance was defined as $p < 0.05$. Abbreviations: OGT, O-linked N-acetylglucosamine transferase; ERK, extracellular signal-regulated kinase; Thr, threonine; Tyr, tyrosine; BMI, body mass index; r, Pearson correlation coefficient.

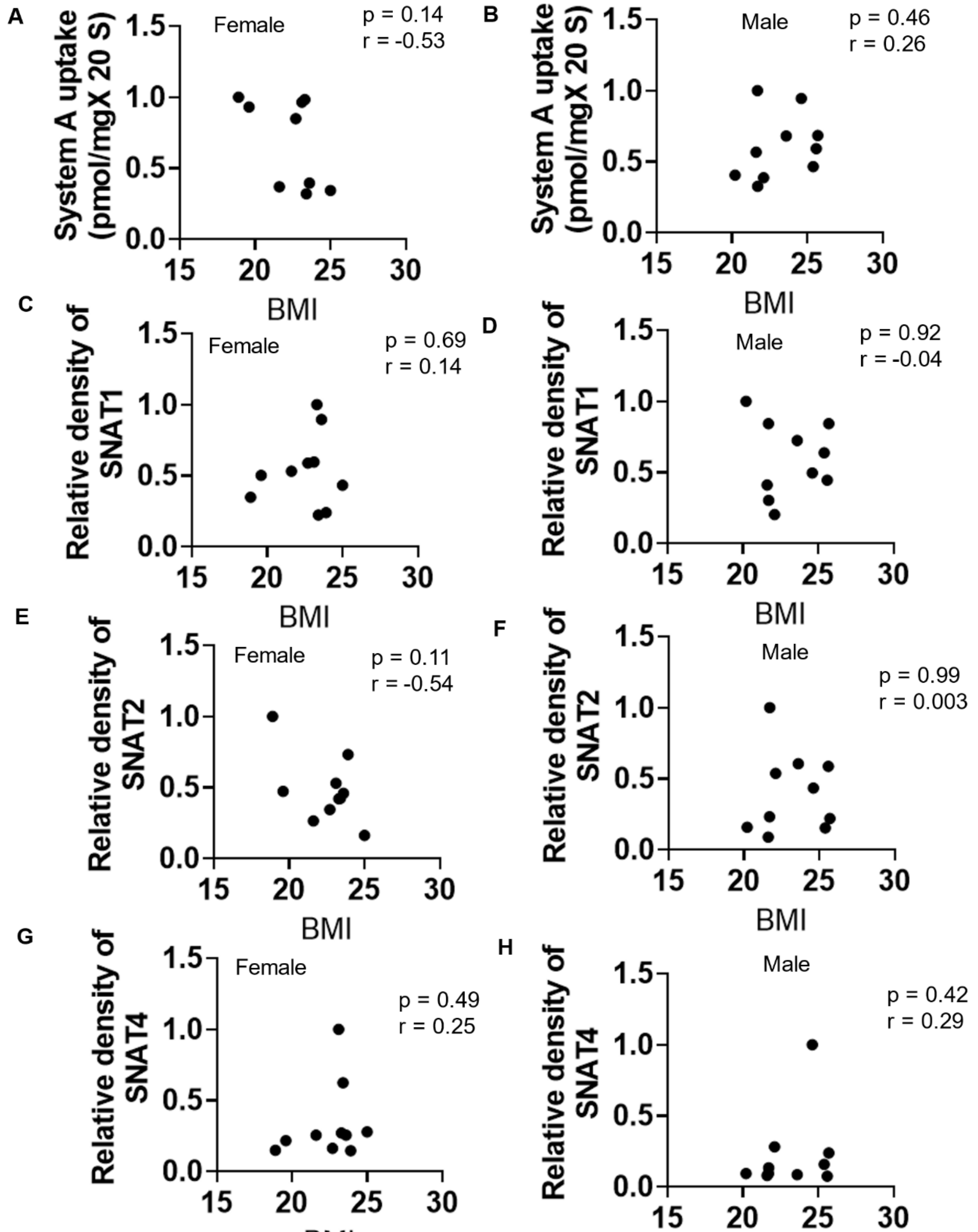

Supplemental Figure S12. Correlation between maternal BMI and System A amino acid transport activity and SNAT protein expression in female and male placentas. Correlation analyses between maternal BMI and System A amino acid transport activity in female (A) and male (B) placentas, SNAT1 protein expression in female (C) and male (D) placentas, SNAT2 protein expression in female (E) and male (F) placentas, and SNAT4 protein expression in female (G) and male (H) placentas. Each symbol represents an individual placenta. Pearson's correlation coefficient (r) and corresponding p value are shown for each analysis. No significant correlations were observed. Values are presented as means ± SEM. Statistical significance was defined as $p < 0.05$. Abbreviations: SNAT, sodium-coupled neutral amino acid transporter; BMI, body mass index; r, Pearson correlation coefficient.

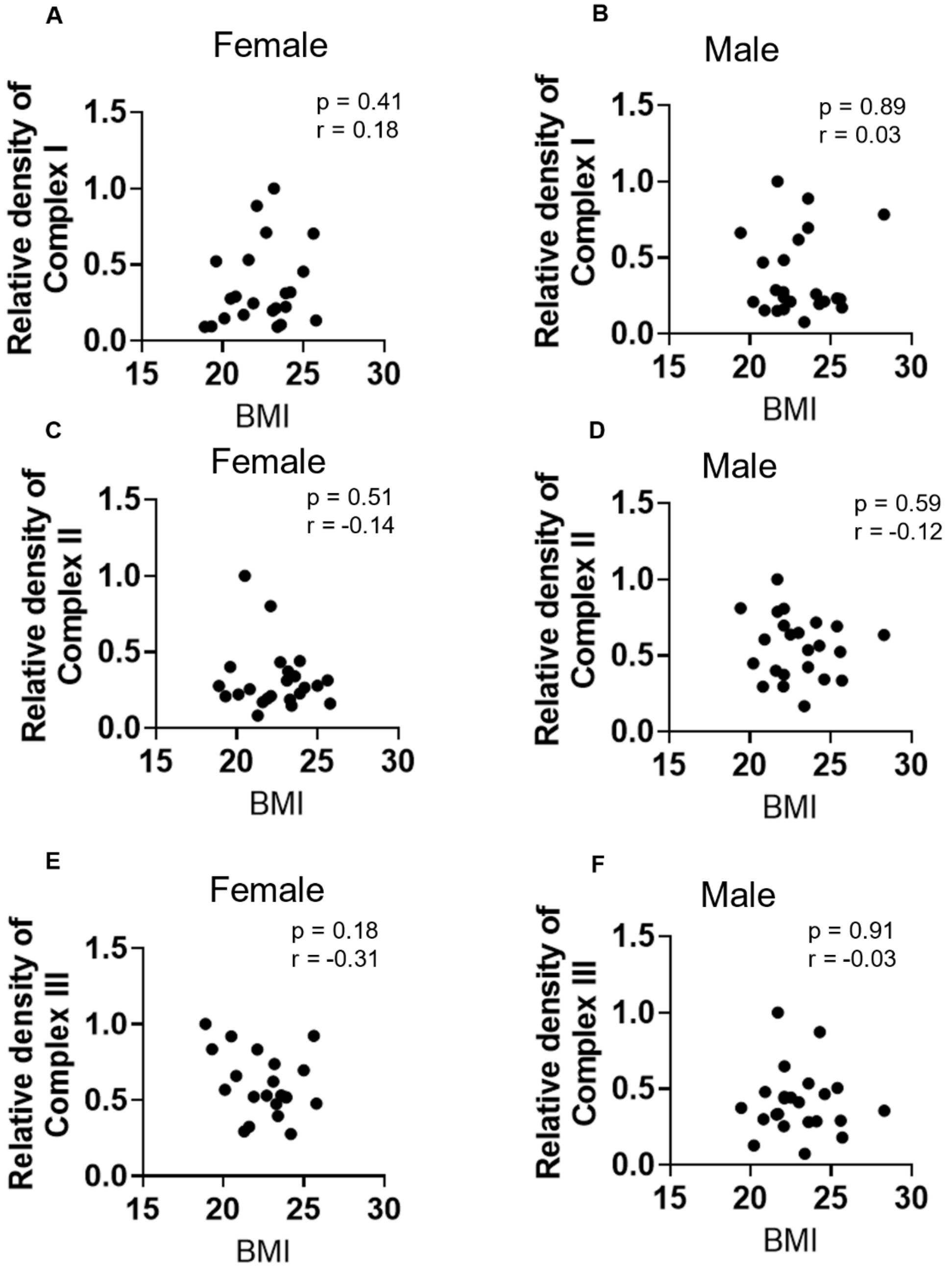

Supplemental Figure S13. Correlation between maternal BMI and mitochondrial electron transport chain complex I–III protein expression in female and male placentas
Correlation analyses between maternal BMI and mitochondrial electron transport chain complex I protein expression in female (A) and male (B) placentas, complex II protein expression in female (C) and male (D) placentas, and complex III protein expression in female (E) and male (F) placentas. Each symbol represents an individual placenta. Pearson's correlation coefficient (**r**) and corresponding **p** value are shown for each analysis. No significant correlations were observed. Values are presented as means ± SEM. Statistical significance was defined as **p < 0.05**. Abbreviations: BMI, body mass index; **r**, Pearson correlation coefficient.

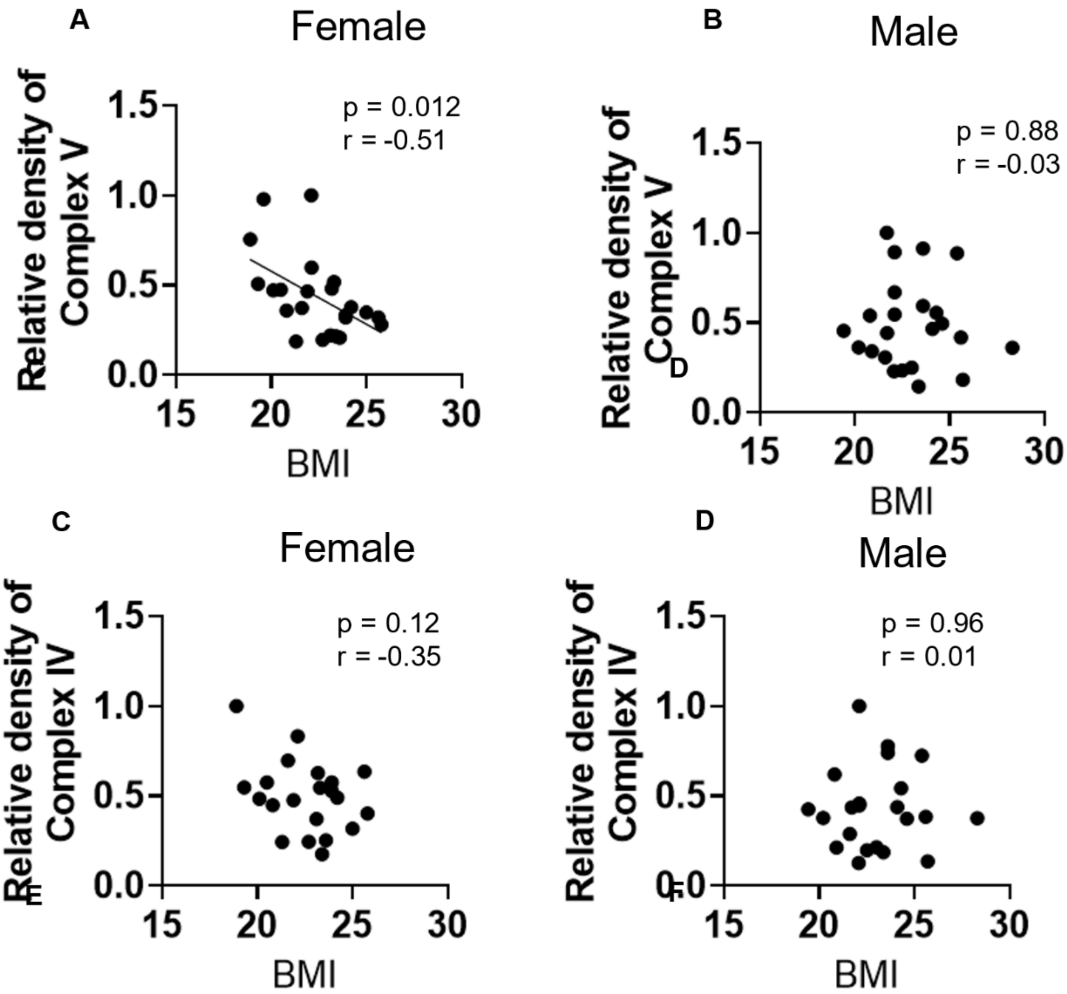

Supplemental Figure S14. Correlation between maternal BMI and mitochondrial electron transport chain complex IV and V protein expression in female and male placentas
Correlation analyses between maternal BMI and mitochondrial electron transport chain complex V protein expression in female (A) and male (B) placentas, and complex IV protein expression in

female (C) and male (D) placentas. Each symbol represents an individual placenta. Pearson's correlation coefficient (**r**) and corresponding **p** value are shown for each analysis. A significant negative correlation between maternal BMI and mitochondrial complex V protein expression was observed in female placentas (A). Values are presented as means ± SEM. Statistical significance was defined as **$p < 0.05$**. Abbreviations: BMI, body mass index; **r**, Pearson correlation coefficient.
